# Interesting Halophilic Sulphur-Oxidising Bacteria with Bioleaching Potential: Implications for Pollutant Mobilisation from Mine Waste

**DOI:** 10.3390/microorganisms11010222

**Published:** 2023-01-15

**Authors:** Chiamaka Belsonia Opara, Nor Kamariah, Jeroen Spooren, Katrin Pollmann, Sabine Kutschke

**Affiliations:** 1 Helmholtz-Zentrum Dresden-Rossendorf, Helmholtz Institute Freiberg for Resource Technology, Bautzner Landstraße 400, 01328 Dresden, Germany; 2 Sustainable Materials Management, Flemish Institute for Technological Research (VITO), Boeretang 200, 2400 Mol, Belgium

**Keywords:** bioleaching, halophilic sulphur-oxidising bacteria, mine waste rock, pollutant mobilisation, *Thiomicrospira cyclica*, *Thiohalobacter thiocyanaticus*, *Thioclava electrotropha*, *Thioclava pacifica*

## Abstract

For many years, research on the microbial-dissolution of metals from ores or waste materials mainly focussed on the study of acidophilic organisms. However, most acidophilic bioleaching microorganisms have limited tolerance to high chloride concentrations, thereby requiring fresh water for bioleaching operations. There is a growing interest in the use of seawater for leaching purposes, especially in regions with less access to fresh water. Consequently, there is a need to find halophilic organisms with bioleaching potentials. This study investigated the bioleaching potentials of four moderately halophilic sulphur-oxidising bacteria: *Thiomicrospira cyclica, Thiohalobacter thiocyanaticus, Thioclava electrotropha* and *Thioclava pacifica*. Results revealed *T. electrotropha* and *T. pacifica* as the most promising for bioleaching. Pure cultures of the two *Thioclava* strains liberated about 30% Co, and between 8–17% Cu, Pb, Zn, K, Cd, and Mn from a mine waste rock sample from the Neves Corvo mine, Portugal. Microwave roasting of the waste rock at 400 and 500 °C improved the bioleaching efficiency of *T. electrotropha* for Pb (13.7 to 45.7%), Ag (5.3 to 36%) and In (0 to 27.4%). Mineralogical analysis of the bioleached residues using SEM/MLA-GXMAP showed no major difference in the mineral compositions before or after bioleaching by the *Thioclava* spp. Generally, the bioleaching rates of the *Thioclava* spp. are quite low compared to that of the conventional acidophilic bioleaching bacteria. Nevertheless, their ability to liberate potential pollutants (metal(loid)s) into solution from mine waste raises environmental concerns. This is due to their relevance in the biogeochemistry of mine waste dumps, as similar neutrophile halophilic sulphur-oxidising organisms (e.g., *Halothiobacillus* spp.) have been isolated from mine wastes. On the other hand, the use of competent halophilic microorganisms could be the future of bioleaching due to their high tolerance to Cl^-^ ions and their potential to catalyse mineral dissolution in seawater media, instead of fresh water.

## 1. Introduction

The extraction and processing of mineral resources generate significant amounts of waste globally, mainly tailings and waste rock. Tailings are a slurry of fine-grained residues resulting from the comminution and beneficiation of ores and typically contain secondary precipitates and processing reagents, such as flotation chemicals [1]. Waste rock consists of low-grade bedrock or earth material excavated during mining operations to access the economically valuable ore and is generally coarse, crushed, or blocky, covering a range of sizes, from very large boulders or blocks to fine sand-size particles [2]. These mine wastes are potential threats to the environment, as their exposure to air and water oxidises the metal sulphides and can result in acid generation and metal(loid) mobilisation into the environment. On the other hand, mine wastes are increasingly being considered as easily accessible reserves for resource recovery due to increased demand for metals, depleting primary mineral ores, deepening mines and increased costs of exploitation. Due to less efficient ore processing in the past, historic mine wastes may have contained higher ore grades than the primary ores currently being mined. In addition, it is often more economically attractive to extract metals from mine wastes which have already been hoisted and partially processed than from deeply buried primary ore deposits [3].

Sulphide mineral oxidation is drastically expedited by iron- and sulphur-oxidising microorganisms, usually acidophilic and chemoautolithotrophic in nature. Biomining, as a biotechnology, utilises these organisms (mainly bacteria and archaea) to extract and recover metal(loid)s from low-grade ores, concentrates and waste materials via bioleaching and biooxidation [4,5]. In comparison to traditional metal extraction methods, such as smelting, biomining has the advantage of cost-effectiveness for low-grade and complex ores. Biomining is also an environmentally friendly alternative due to its low atmospheric emissions and energy demands [6]. Biomining has been applied successfully in the bioleaching of Cu, Zn, Ni, Co, uranium and biooxidation of refractory gold ores [7]. More than 30 species have been identified as playing direct and indirect roles in biomining, and the crucial reason for their success is that they operate as microbial consortia and not independently [8]. A stable and efficient sulphide mineral degradation is ensured by the synergistic effect of these microbial consortia, often consisting of three major groups of acidophilic prokaryotes (i) the Fe-oxidising organisms (oxidant manufacturers), which oxidise ferrous iron to ferric iron, the main oxidant of sulphide minerals; (ii) the sulphur-oxidising organisms (acid generators), which sustain the optimum acidity levels for the consortia by oxidising reduced sulphur and generating sulphuric acid (iii) the janitors, which break down organic carbon compounds released from living and dead cells, thereby preventing their accumulation and inhibition of other living cells [9]. A specific organism may play one or more of these roles in the bioleaching consortia [10].

Acidophilic bioleaching organisms, especially the iron-oxidising organisms, tend to be sensitive to chloride ions [11]. Therefore, the application of biomining is constrained in regions with extremely high chloride content of soils and source waters, such as Chile, where access to fresh water for mineral processing is scarce and becoming an economic limitation [12]. This has sparked intense interest in finding halotolerant microorganisms that can actively bioleach in seawater media [13]. The most widely studied organisms in bioleaching, such as *Acidithiobacillus ferrooxidans, Leptospirillum ferriphilum* and *Sulfobacillus thermosulfidooxidans* were shown to be inhibited at 6, 12, and 12 g/L of NaCl, respectively [14,15,16]. Chloride ion stress alters the mechanism by which acidophilic bioleaching organisms tolerate low pH [17]. The chloride anion can cross the bacterial cell membrane, resulting in an influx of protons, acidification of the cytoplasm, reduced metabolic activity and finally, cell death [18,19]. Only a small number of microbes that can withstand both high osmotic stress and low pH at the same time have been isolated, due to the fact that environments where both stresses co-exist are uncommon, such as volcanoes near seawater and acidic lakes and drains. A mixed culture dominated by *Acidihalobacter* spp. and enriched from shallow acidic pools at the Aeolian Islands, Vulcano, Italy, and pure cultures of *Acidihalobacter prosperus* (DSM 14174) and *Acidihalobacter ferrooxidans* (DSM 14175) were able to oxidise pyrite at 30 g/L of chloride concentration [17].

Sulphur-oxidising bacteria play important roles in various environmental and industrial settings. They are capable of utilising inorganic sulphur compounds as their energy source, including sulphides, sulphite (SO_3_^2−^), elemental sulphur (S_0_) and thiosulphate (S_2_O_3_^2−^) [20]. They produce sulphuric acid as their final product, an inorganic acid that has a strong degrading action [21]. The bioleaching potential of neutrophilic–halophilic sulphur-oxidising bacteria has not been studied extensively, despite being potentially useful for bioleaching processes in salt-containing environments. The aim of this study was to assess the bioleaching potential of four halophilic and neutrophilic/alkaliphilic sulphur-oxidising bacteria (*Thiomicrospira cyclica, Thiohalobacter thiocyanaticus, Thioclava electrotropha* and *Thioclava pacifica*), in the mobilisation of both valuable and hazardous metal(loid)s from the Neves Corvo waste rock (NC_01), at moderate to high chloride concentrations.

## 2. Materials and Methods

### 2.1. Origin, Preparation and Characterisation of the Mine Waste Sample

The Neves Corvo mine, Portugal, is an underground Cu-(Sn)-Zn mine located within the western portion of the prominent Iberian Pyrite Belt (IPB) [22]. The mine generates two main types of extractive waste residues: waste rock and tailings, which are sub-aerially co-deposited in the 190 ha Cerro do Lobo tailings management facility (TMF) [23]. Between 2010 and 2019, the mine accumulated 17 Mt of thickened tailings and 7.3 Mt of waste rock at the TMF, while 3.1 Mt of waste rock was deposited in a temporary stockpile [24].

A freshly hoisted waste rock (NC_01) sample was obtained from the Neves Corvo mine. In preparation for the bioleaching experiments, NC_01 was dried at 40 °C and milled to <100 µm particle size using a Retsch jaw crusher BB 200 and a Retsch RS 200 vibratory disc mill. NC_01 was acid-digested with a mixture of HNO_3_ (Roth p.a., 65%) + HCl (Merck p.a., 30%) and HF (Roth suprapur, ~48%) in a ratio of 1:3:1 in a microwave (Multiwave 3000, Anton Paar GmbH, Graz, Austria) at 240 °C and 800 W for 10 min. Then, the chemical composition was analysed using inductively coupled plasma mass spectrometry (ICP-MS) (NexION 350×, 1300 Watts, Argon plasma gas, Perkin Elmer, Waltham, MA, USA). As shown in Table 1, the waste rock was mainly composed of 1800 mg/kg of Cu, 800 mg/kg of Pb, 3600 mg/kg of Zn, and 104,500 mg/kg of Fe.

The mineral composition of NC_01 was determined by a scanning electron microscope-based automated image analysis technique called a Mineral Liberation Analyzer (MLA). The MLA system is able to collect quantitative mineralogical and textural data for samples. The MLA consisted of: (i) a Quanta 650F field emission scanning electron microscope (FE-SEM) (FEI, Hillsboro, OR, USA), (ii) two Quantax X-Flash 5030 energy-dispersive X-ray (EDX) detectors (Bruker, Billerica, MA, USA), and (iii) the MLA software suite version 3.1. To prepare the samples for MLA, 3 g of each sample was mixed with graphite powder and embedded in 25 mm epoxy resin, which was cut vertically, rotated at 90 °C, re-embedded into a second epoxy-resin and then polished to obtain a 30 mm grain mount. The grain mount (containing the sample) was carbon-coated and measured by MLA using the GXMAP measurement mode at horizontal frame width of 500 pixels, 4.78 spot size, probe current of 10 nA, and acceleration voltage of 25 kV.

All chemicals and reagents used in this study were of analytical grade.

### 2.2. Organisms and Cultivation Conditions

Four moderately halophilic, sulphur-oxidising bacteria were used in this study, namely, *Thiomicrospira cyclica* DSM-14477, *Thiohalobacter thiocyanaticus* DSM-21152, *Thioclava electrotropha* DSM 103712^T^ and *Thioclava pacifica* DSM-10166. They were all obtained from the German Collection of Microorganisms and Cell Cultures (DSMZ). *T. cyclica* is obligately chemolithoautotrophic and was originally isolated from the hypersaline and alkaline Mono lake in Mono County, CA, USA [25]. Cultivation was performed with a sterilized mineral medium (pH = 10) containing in g/L: Na_2_CO_3_ 21, NaHCO_3_ 9, NaCl 5, K_2_HPO_4_ 0.5, supplemented with 40 mM Na_2_S_2_O_3_·5H_2_O and 5 mM KNO_3_. *T. thiocyanaticus* is also obligately chemolithoautotrophic and was originally isolated from sediments in hypersaline chloride–sulphate lakes in the Kulunda Steppe, in south-western Siberia, Russia [26]. Cultivation of *T. thiocyanaticus* was performed with a sterile mineral medium (pH = 7.5) containing in g/L: NaCl 60, K_2_HPO_4_ 1.5, NH_4_Cl 0.5, CaCl_2_·2H_2_O 0.05, MgSO_4_·7H_2_O 0.5, Na_2_S_2_O_3_·5H_2_O 5, and NaHCO_3_ 5.

*T. electrotropha* and *T. pacifica* are both facultative autotrophs originally isolated from Catalina Harbor in California (USA) [27] and a sulphidic hydrothermal area in Matupi Harbour in New Britain, Papua New Guinea [28], respectively. Pure cultures of both *Thioclava* spp. were first cultivated heterotrophically in a sterile LBI medium (pH = 7) containing in g/L: typtone 10, yeast extract 5, NaCl 20, MgCl_2_.6H_2_O 3, and CaCl_2_.2H_2_O 0.15. Then, they were sub-cultured for autotrophic growth into sterilised artificial seawater (ASW) medium (pH = 6.5) containing in g/L: NaCl 20, MgCl_2_·6H_2_O 3, CaCl_2_·2H_2_O 0.015, KCl 0.5, NH_4_Cl 0.535, KH_2_PO_4_ 0.136, Na_2_SO_4_ 0.142, and Na_2_S_2_O_3_·5H_2_O 6.21. All cultures were incubated at room temperature in 100 mL Erlenmeyer flasks on a rotary shaker (120 rpm).

### 2.3. Bioleaching Experiments

The bioleaching potentials of the four halophilic sulphur-oxidising bacteria were determined by incubating pure cultures of each organism with the waste rock sample in 250 mL Erlenmeyer flasks, and constantly agitated at 160 rpm at room temperature. The bioleaching experiments were carried out at 5 wt% solid content, using 10% *v/v* inoculum from cultures containing between 10^9^–10^10^ CFU/mL. The same mineral medium used to culture each organism (Section 2.2) was also used for bioleaching. The final volume of the liquid phase in the bioleaching flasks was 100 mL.

For each organism, one-step, two-step and spent medium bioleaching studies were conducted. In one-step bioleaching (1SB), each organism was inoculated into the mineral medium containing the waste rock sample. In two-step bioleaching (2SB), the organisms were allowed to grow to the exponential stage before the addition of the waste rock sample on day 2. In spent medium bioleaching (SMB), the bacterial cells were separated from the liquid media after maximum growth of the organisms prior to using the liquid media as lixiviants for the leaching of the waste rock. The 2SB was conducted to investigate if the contents of the waste rock were toxic to the growth or bioleaching capacity of the organisms. Meanwhile, the SMB was conducted to investigate if the mechanism of bioleaching involved an indirect process that only requires the metabolic products of the organisms.

There was an abiotic control where an equal volume of deionised water substituted the total volume of the mineral medium and the inoculum. Sampling of the leachate was performed every 7 days to monitor the pH (Mettler Toledo), sulphate concentration (Thermo Scientific (San Jose, CA, USA) Dionex Integrion high-performance ion chromatography) and soluble metal(loid) concentrations (Perkin Elmer, ICP-MS). All experiments were run in duplicate for 28 days, and the mean values are reported. Metal(loid) recovery after n days of incubation was calculated as follows:

$$\%\;\mathrm{metal}\left(\mathrm{loid}\right)\;\mathrm{recovery}=\frac{\mathrm{Mn}-\mathrm{Mi}}{\mathrm{Mt}}\times 100$$

where Mi is the initial mass of the element in the liquid mineral medium, Mn is the mass of the element in the leachate on day-n, and Mt is the total mass of the element in the solid waste sample. At the end of the bioleaching experiments, bioleached residues from promising organisms were harvested, dried at room temperature and then analysed for their chemical and mineralogical content, using the same conditions stated in Section 2.1.

### 2.4. Bioleaching Optimisation Experiments

After the initial screening of the four organisms for bioleaching potential, various processes that may affect the bioleaching activities were studied with the aim of improving the bioleaching activities of the promising organisms. Bioleaching conditions were the same as in Section 2.3, unless otherwise stated. These optimisation processes include:

(i) Effect of the *Thioclava* consortium on the bioleaching of NC_01

*T. electrotropha* and *T. pacifica* were grown together to obtain a *Thioclava* consortium which was used at 10% *v/v* to conduct 1SB, 2SB and SMB of NC_01 at 5% solid content for 28 days.

(ii) Effect of weekly serial addition of NC_01 and partial nutrient replacement on the bioleaching of NC_01

1SB and 2SB were conducted for the two *Thioclava* spp. using 10% *v/v* inoculum. NC_01 was added weekly to obtain 1%, 3% and 5% solid content for week 0, week 1 and week 2, respectively. Half of the leachate was replaced weekly with a fresh mineral medium. This was conducted by centrifuging half of the leachate, discarding the supernatant and resuspending the solids in a fresh mineral medium before transferring it back to the bioleaching conical flasks. The experiment was run for 42 days.

(iii) Effect of high temperature and pressure on the bioleaching of NC_01

The two *Thioclava* spp. were grown in the ASW medium for 21 days. Then, the spent medium (pH ≈ 3.1) was used as lixiviants for the bioleaching of NC_01 at high temperature (121 °C) and pressure (103,421.4 pa) for 30 min, using an autoclave (Steriltechnik AG, Haldensleben, Germany). After cooling, leachates were analysed for dissolved metal concentrations using the ICPMS.

(iv) Effect of microwave roasting on the bioleaching of NC_01

NC_01 samples were placed in alumina crucibles and heated in a ventilated PYRO advanced microwave furnace (Milestone, Shelton, CT, USA) at 400, 500 and 600 °C, at a ramp time of 30 min and a dwell time of 60 min. The major mineral phases of the microwave-roasted samples were determined using X-ray diffraction (XRD) (PANalytical Empyrean diffractometer, Malvern, United Kingdom [Co-tube, 2 theta 5–80° with a step size of 0.013°, 35 kV and 35 mA, PIXcel 3D medipix 1 × 1 area detector]). 1SB and 2SB of the microwave-roasted NC_01 were then conducted with *T. electrotropha* for 14 days at 5% solid content, using 10% *v/v* inoculum.

## 3. Results

### 3.1. Bioleaching Results of the Four Halophilic Sulphur-Oxidising Bacteria

**Figure 1:**
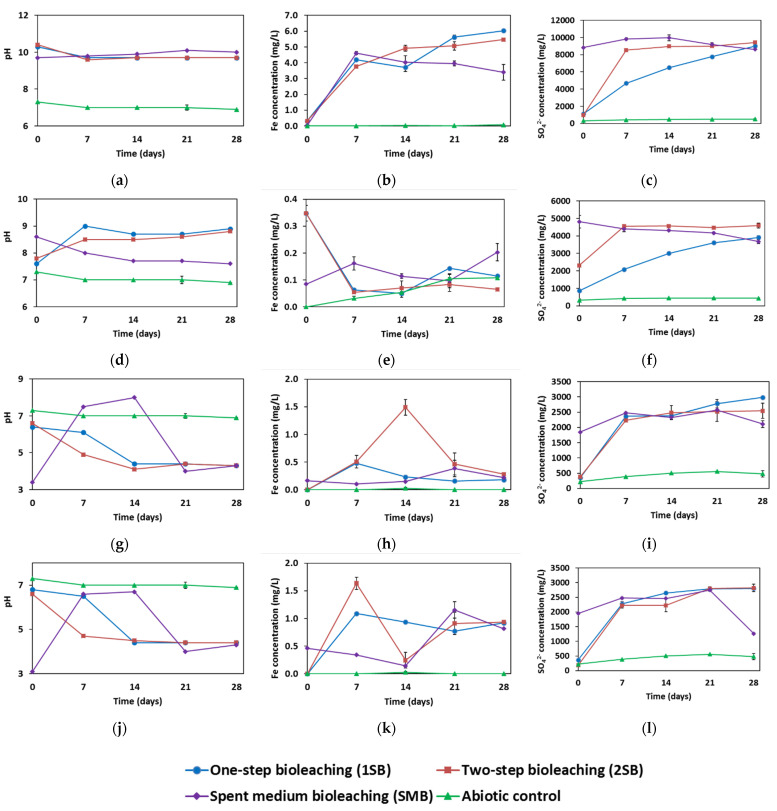
The evolution of pH, Fe and sulphate (SO_4_^2−^) concentrations during the bioleaching of the Neves Corvo waste rock (NC_01) by *T. cyclica* (**a**–**c**)*, T. thiocyanaticus* (**d**–**f**)*, T. electrotropha* (**g**–**i**) and *T. pacifica* (**j**–**l**). Values are the average of duplicate flasks ± standard deviations.

The concentrations and percentages of metal(loid)s extracted from NC_01 by *T. cyclica, T. thiocyanaticus, T. electrotropha* and *T. pacifica* are presented in Tables S1 and Table A1, Tables S2 and Table A2, Tables S3 and Table A3, and Tables S4 and Table A4 (Supplementary Material and Appendix A), respectively. The evolution of pH, total dissolved Fe and sulphate concentrations during the four bioleaching experiments are presented in Figure 1. An increase in sulphate concentration arising from the ability of the four halophilic organisms to oxidise reduced sulphur indicated bacterial growth and activity during the bioleaching experiments. A decreasing solution pH was also used as an indication of sulphur oxidation, and therefore, bacterial growth and activity, as pH reduces with an increasing sulphate concentration [13]. Biomass growth and activity in the presence of the waste rock indicated that the organisms were tolerant to the high metal(loid) contents of the waste rock. The bioleaching capability of the halophilic organisms was assessed based on the quantity of dissolved metal(loid)s (Cu, Pb, Zn, Co, As, Cd, K, Sb, Ag and Mn) in solution resulting from the oxidation of sulphide minerals in NC_01. Generally, for all four organisms, no bioleaching assay (1SB, 2SB and SMB) was observed to be consistently more efficient. The extraction efficiencies for 1SB, 2SB and SMB were comparable for most meta(loid)s.

Sulphur oxidation by *T. cyclica* produced sulphuric acid, thereby drastically increasing the SO_4_^2−^ concentration and reducing the solution pH within the first 7 days. Specifically, there was a steep increase in sulphate concentration from its initial concentration (~1000 mg/L) on day 0 to 5000 mg/L (for 1SB) and 8535 mg/L (for 2SB) on day 7. The SO_4_^2−^ concentration continued to increase, reaching 9000 mg/L by the end of the 1SB and 2SB experiments. The pH decreased from 10.3 to 9.7 within the first 7 days during the exponential growth phase of *T. cyclica* and remained relatively constant until the end of the 1SB and 2SB experiments. The oxidation of reduced sulphur compounds during the bioleaching of NC_01 by *T. cyclica* resulted in metal(loid) dissolution. The total dissolved Fe was also observed to increase within the first 7 days of 1SB and 2SB, from 0.3 to 4.2 mg/L, and continued to increase until around 5.7 mg/L on the last day. By the end of the bioleaching experiment with *T. cyclica*, (%) 6.0 Cu, 0.6 Pb, 0.8 Zn, 6.2 Co, 44.2 As, 2.3 Cd, 17.4 K, 38.7 Sb, 23.7 Ag, and 0.0 Mn were solubilised from NC_01 via 1SB, 2SB and/or SMB (the highest recovery among the three strategies is stated). However, the maximum recovery for some elements was attained before the last day. For example, Cu and K recoveries reached a maximum (12.5% and 31.2%, respectively) on day-7 via 1SB.

*T. thiocyanaticus* dissolved (%) 7.3 Cu, 0.4 Pb, 0.1 Zn, 1.3 Co, 0.7 As, 3.3 Cd, 5.2 K, 25 Sb, 14.2 Ag, and 0.3 Mn from NC_01 within the first 7 days. After day 7, the recoveries either remained constant or decreased, except for K, Sb and Ag, which increased to 7.2, 38.7 and 31.8%, respectively, towards the end of the experiment. The pH increased from 7.8 to 9 and 8.5 in 1SB and 2SB, respectively, within the first 7 days, with no major change after this. On the other hand, there was less than a 2-fold increase in sulphate concentrations in the bioleaching assays. The SO_4_^2−^ concentration rose from 2315 to 4560 mg/L in the first 7 days and remained constant until the end of the 2SB experiment. While for 1SB, the SO_4_^2−^ concentration increased gradually throughout the duration of the experiment from 863 mg/L on day 0 to 3915 mg/L on the final day. The pH and SO_4_^2−^ concentration in SMB decreased slightly through the experiment, from 8.6 to 7.6 and 4815 to 3675 mg/L, respectively. The total dissolved Fe decreased considerably within the first 7 days from its initial concentration (0.3 g/L), reaching 0.06 g/L on day 7, and then remained below 0.2 till the end of the 1SB and 2SB experiments.

Bacterial growth and activity led to a marked decrease in pH from 6.5 to 4.3 within the first 7 days for 2SB and the first 14 days for 1SB, and then remained constant till the end of the experiments with *T. electrotropha*. The SO_4_^2−^ concentration first increased almost 7-fold (from ~350 to 2300 g/L) within the first 7 days, and then gradually till day 28 reaching 2985 mg/L for 1SB and 2545 mg/L for 2SB. Sulphide minerals were oxidised during the bioleaching of NC_01 by *T. electrotropha*. The total dissolved Fe increased from 0 to 0.5 mg/L within the first 7 days but remained below 0.6 mg/L throughout the duration of all the bioleaching assays. Except for 2SB, where it reached 1.5 mg/L on day 14 but went back to 0.5 g/L by the 21st day. The percentage of metal(loid)s extracted by *T. electrotropha* generally increased with time. By day 28, *T. electrotropha* leached (in %) 7–8.7 Cu, 9.6–12.4 Pb, 10.3–12.8 Zn, 23.5–24.9 Co, 2.1–4.8 As, 13.7–16.9 Cd, 0–21 K, 1.6–2.1 Sb, 3.6–5.2 Ag, and 12.7–13.9 Mn, from NC_01 via 1SB, 2SB and SMB.

The percentages of metal(loid)s solubilised by *T. pacifica* was comparable to that of *T. electrotropha*. At the end of the experiment, *T. pacifica* extracted (in %) between 5–8.1 Cu, 9.9–13 Pb, 9.4–12.6 Zn, 24.1–26.7 Co, 1.7–2.7 As, 13.1–16.8 Cd, 0–9.3 K, 1.1–2.2 Sb, 2.9–4.4 Ag, and 11.3–13.3 Mn from NC_01 via 1SB, 2SB and SMB. As in the bioleaching experiment with *T. electrotropha*, the percentage of metal(loid)s extracted by *T. pacifica* also generally increased with time. There was a steep increase in the total dissolved Fe in 1SB and 2SB within the first seven days, from 0 to 1.1 mg/L for 1SB and 1.6 mg/L for 2SB. This preceded a steep decrease to 0.2 mg/L by day 14 for 2SB and another increase by day 21 to 0.9 mg/L, before remaining constant till the end of the experiment. For 2SB, the Fe concentration decreased gradually after the 7th day, reaching 0.9 mg/L on the last day. The evolution in pH and SO_4_^2−^ concentration in all the bioleaching assays were similar to that of *T. electrotropha*. A decrease in pH and an increase in SO_4_^2−^ concentration during 1SB and 2SB indicated the growth and activity of *T. pacifica*.

Little or no sulphur oxidation occurred in the abiotic controls. The pH remained within the neutral range throughout the experiment. The SO_4_^2−^ concentration was also constant and remained below 500 mg/L. The total dissolved Fe was either absent or very low, at less than 0.1 mg/L. In comparison to all the bioleaching assays, there was little or no metal(loid) extracted in the abiotic control assays. One exception was Mn, where the extraction efficiency of *T. cyclica* and *T. thiocyanaticus* was 7% higher than in the abiotic controls, whereas a lower equivalent extraction efficiency was recorded with *T. electrotropha* and *T. pacifica*.

**Figure 2:**
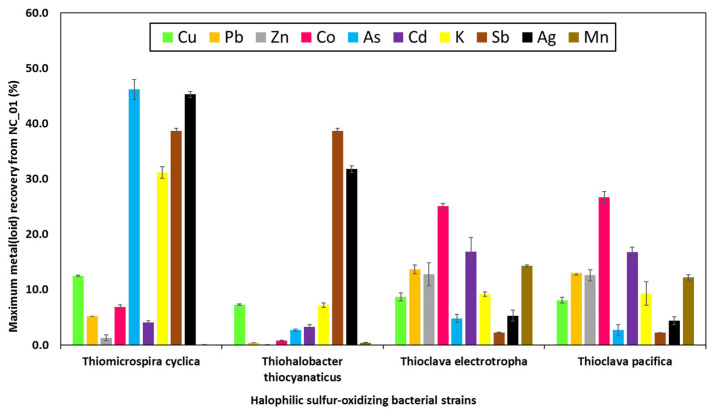
Maximum percentages of metal(loid)s extracted from the Neves Corvo waste rock (NC_01) by four halophilic sulphur-oxidising bacterial strains. Values are the average of duplicate flasks ± standard deviations.

Figure 2 compares the maximum metal(loid) recoveries of the four halophilic sulphur-oxidising bacteria. While *T. cyclica* mobilised substantial amounts of As (46.2%), K (31.2%), Sb (38.7%) and Ag (45.3%), the two *Thioclava* species both mobilised a wider range of metal(loid)s into solution. *T. electrotropha* and *T. pacifica* solubilised between 8–17% of the total Cu, Pb, Zn, Cd, K, and Mn contents of NC_01, as well as 25–27% Co. Except for Sb and Ag with good recoveries (32–39%), the maximum percentages of other elements extracted by *T. thiocyanaticus* were below 7%.

### 3.2. Bioleaching Optimisation Results

Due to the bioleaching potential observed for *T. electrotropha* and *T. pacifica*, three optimisation processes were attempted to improve their extraction efficiencies, and a fourth study was conducted with a consortium of both microorganisms.

#### 3.2.1. Results of the Bioleaching of NC_01 by the *Thioclava* Consortium

**Figure A1:**
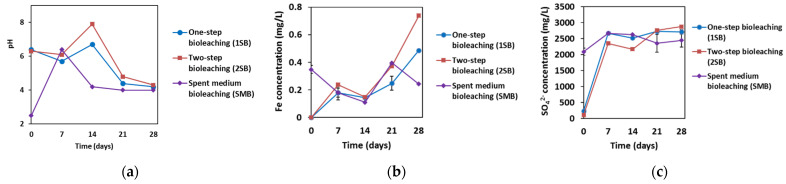
The evolution of pH (**a**), Fe (**b**) and sulphate (SO_4_^2−^) (**c**) concentrations during the bioleaching of the Neves Corvo waste rock (NC_01) by the *Thioclava* consortium. Values are the average of duplicate flasks ± standard deviations.

The concentrations and percentages of metal(loid)s extracted from NC_01 by the *Thioclava* consortium containing *T. electrotropha* and *T. pacifica* are presented in Table S5 and Table A5; while the evolution of pH, total dissolved Fe and SO_4_^2−^ concentration are presented in Figure A1. Compared to the non-optimised bioleaching experiments with *T. electrotropha* and *T. pacifica*, there was no major improvement in the extractions of Cu, Zn, As, Sb, Ag and Mn. However, the extractions (in %) of Pb increased slightly from 13.7 ± 0.8 to 18.6 ± 0.8, and K from 9.2 ± 0.4 to 19.6 ± 1.3 via SMB.

#### 3.2.2. Effects of Weekly Serial Addition of NC_01 and Partial Nutrient Replacement on the Bioleaching of NC_01

**Figure A2:**
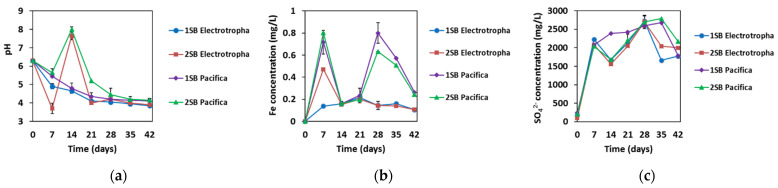
The evolution of pH (**a**), Fe (**b**) and sulphate (SO_4_^2−^) (**c**) concentrations during a process optimisation experiment (weekly serial addition of NC_01 and partial nutrient replacement) via one-step bioleaching (1SB) and two-step bioleaching (2SB) of the Neves Corvo waste rock (NC_01) via by *T. electrotropha* and *T. pacifica*. Values are the average of duplicate flasks ± standard deviations.

Table S6 and Table A6 present the concentrations and percentages of metal(loid)s extracted from NC_01 by *T. electrotropha* and *T. pacifica* with the serial addition of NC_01 and partial nutrient replacement. Whereas the evolution of pH, total dissolved Fe and SO_4_^2−^ concentration are presented in Figure A2. The results showed that the percentages of the extracted Pb, Co, As, K, Ag and Mn increased slightly in comparison to the non-optimised experiments. Extractions (in %) of Co increased from 25.1 ± 0.5 to 31.1 ± 0.1, As from 4.8 ± 0.7 to 7.1 ± 0.3, K from 9.2 ± 0.4 to 19 ± 0, Ag from 5.3 ± 1 to 11.6 ± 1.2 and Mn from 14.3 ± 0.2 to 18.4 ± 0.9 via 1SB and/or 2SB.

#### 3.2.3. Results of the Bioleaching of NC_01 at High Temperature and Pressure

Table A7 presents the concentrations and percentages of metal(loid)s extracted from NC_01 at high temperature and pressure using the spent medium of *T. electrotropha* and *T. pacifica*. While the pH and SO_4_^2−^ concentration before and after high temperature and pressure leaching are presented in Table A8. Only the extractions of Ag and Mn improved when NC_01 was leached at high temperature and pressure by the spent media of the two *Thioclava* spp. Compared to the non-optimised experiments, the recovery (%) of Ag increased from 5.3 ± 1 to 10 ± 0.9 and 11.4 ± 0.3, while that of Mn increased from 14.3 ± 0.2 to 23.2 ± 0 and 24.8 ± 0.1, at high temperature and pressure, by *T. electrotropha* and *T. pacifica*, respectively. Additionally, 2.3% ± 0.1 In was extracted by the spent medium of *T. pacifica*.

#### 3.2.4. Results of the Microwave-Assisted Bioleaching of NC_01

**Figure 3:**
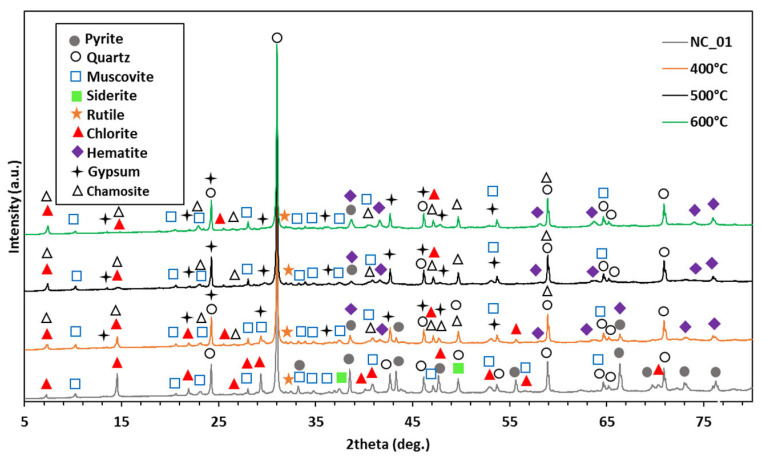
XRD scans of the Neves Corvo waste rock (NC_01) before and after microwave roasting at 400, 500, and 600 °C.

XRD analysis revealed that some minerals in NC_01 (e.g., pyrite) decreased after microwave roasting, while some new minerals (e.g., hematite) were formed. The diffraction intensity peaks of some minerals disappeared or decreased in the XRD diffractograms (Figure 3) while new peaks were formed, indicating that the initial minerals were converted into other minerals.

**Figure A3:**
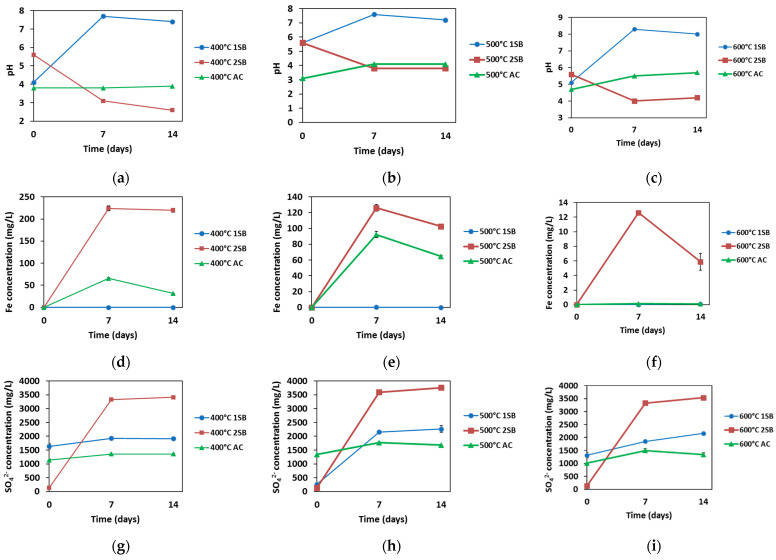
The evolution of pH (**a**–**c**), Fe (**d**–**f**) and SO_4_^2−^ (**g**–**i**) concentrations during the bioleaching of the microwave-roasted (at 400, 500 and 600°C) Neves Corvo waste rock (NC_01) by *T. electrotropha* via one-step bioleaching (1SB), two-step bioleaching (2SB) and the abiotic control (AC). Values are the average of duplicate flasks ± standard deviations.

Table S7 and Table A9 present the concentrations and percentages of metal(loid)s extracted from microwave-roasted NC_01 at 400, 500, and 600 °C by *T. electrotropha*. At the same time, the evolution of pH, total dissolved Fe and SO_4_^2−^ concentration are presented in Figure A3. The extractions of most metal(loid)s from NC_01 improved greatly after microwave roasting. However, this improvement was not specific to the bioleaching assays, but was also observed for the abiotic controls (with deionised water). For example, 46.2 and 49.3% Cu were extracted from the 400 °C-microwave-roasted NC_01 by 1SB and 2SB, respectively, by day 7. While 52% Cu was extracted by deionised water (abiotic control) on the same day. Nevertheless, the extractions of Pb, Ag and In were not comparable to that of the abiotic controls. Compared to the non-optimised experiments, the extraction efficiencies of Pb, Ag and In from microwave-roasted NC_01 increased from (%) 13.7 ± 0.8 to 45.7 ± 0.3, 5.3 ± 1 to 36 ± 2.6 and 0 to 27.4 ± 0.5, respectively. Additionally, *T. electrotropha* recovered greater amounts of Pb, Ag and In from the NC_01 samples roasted at 400 and 500 °C than the samples roasted at 600 °C. For example, the maximum percentage Pb extracted from NC_01 roasted at 400, 500, and 600 °C were 45.7, 41 and 28.8%, respectively.

Contents of the microwave-roasted NC_01 were observed to be toxic to the growth of *T. electrotropha* and therefore affected the bioleaching capacity of *T. electrotropha*. Unlike in the 2SB experiments, little or no sulphur oxidation occurred in the 1SB experiments. Instead, there was an increase in pH and little or no increase in SO_4_^2−^ concentrations within the first 7 days. The pH in the 1SB experiments increased within the first 7 days from around 4 and 5 on day 0 to 8 on day 7. In contrast, there was a marked decrease in pH and an exponential increase in SO_4_^2−^ concentrations in all 2SB experiments within the first 7 days. The pH in the 2SB experiments decreased from 5.6 on day 0 to between 3 and 4 on day 7. While SO_4_^2−^ concentrations increased 24-fold from its initial concentration (136 mg/L) on day 0 to over 3300 mg/L by day 7. Additionally, most of the elements, especially Pb and Ag, were only recovered via the 2SB, and not 1SB.

**Figure 4:**
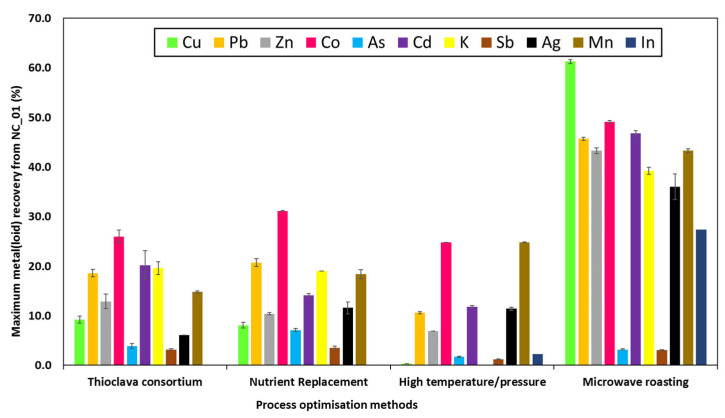
Maximum percentage of metal(loid)s extracted from the Neves Corvo waste rock (NC_01) by the *Thioclava* spp. via four process optimisation methods. Values are the average of duplicate flasks ± standard deviations.

As seen in Figure 4, microwave-assisted bioleaching of NC_01 by *T. electrotropha* was the most efficient process optimization method.

### 3.3. Mineralogical Analysis of NC_01 and the Bioleached Residues by T. electrotropha and T. pacifica

Results from the SEM/MLA-GXMAP showed that the waste rock was mainly composed of silicate and sulphide minerals. NC_01 contained (wt%) 38 quartz, 15 muscovite, 25.9 of other silicate minerals, 15.2 pyrite, 0.1 arsenopyrite, 0.6 chalcopyrite, 0.04 galena, 0.6 sphalerite, 0.04 cobaltite, 0.01 stannite, 2.6 carbonates (siderite, ankerite, calcite, dolomite, magnesite), 0.4 sulphates (gypsum) and 1.34 oxides (rutile, Mg-ilmenite, corundum, cassiterite, Fe-oxide) minerals. The MLA also estimated how elements were distributed across different minerals (Table A10). For example, 91.2% of the total Cu content of NC_01 was hosted by chalcopyrite. Surprisingly, Pb was mainly distributed between oxides (45.7%), galena (36.9%) and pyrite (10.1%). Furthermore, 96.4% of Zn was hosted by sphalerite while 94% of sulphur was hosted by pyrite. Fe was distributed between pyrite (58.2%), silicate (26.3%), carbonates (8.1%) and oxides (5.4%). Some elements were associated with pyrite, e.g., 10.1% Pb, 3.6% Zn, 16% Co and 15.5% Mn.

The bioleached residues from the bioleaching experiments with the two promising bacteria (*Thioclava* spp.) were analysed for changes in mineral compositions. Table A11 compares the mineralogy of NC_01 before and after bioleaching by *T. electrotropha* and *T. pacifica*. Very slight (and sometimes inconsistent) changes were detected in the mineral compositions of NC_01 after bioleaching. Pyrite decreased from 15.5 ± 0.3 wt% to 14.3 ± 0.3 wt% after bioleaching by *T. pacifica*, but remained relatively the same (15.8 ± 0.4 wt%) after bioleaching by *T. electrotropha*. The total concentration of the other sulphide minerals was comparatively the same, from 1.4 ± 0.1 wt% before bioleaching, to 1.6 ± 0.5 and 1.3 ± 0 wt% after bioleaching by *T. electrotropha* and *T. pacifica*, respectively. The total concentration of oxide minerals increased from 1.3 ± 0.2 wt% to 1.9 ± 0 wt% and 1.7 ± 0.1 wt% after bioleaching by *T. electrotropha* and *T. pacifica*, respectively. For the sulphate minerals, gypsum (0.4 wt%) in NC_01 disappeared after bioleaching, while small quantities of jarosite (~0.1 wt%) were formed in the bioleached residues. The wt% of other minerals (groups) remained approximately the same after bioleaching.

## 4. Discussion

The aim of this study was to investigate the capacity of four halophilic sulphur-oxidising bacteria (*Thiomicrospira cyclica, Thiohalobacter thiocyanaticus, Thioclava electrotropha* and *Thioclava pacifica*) to mobilise metal(loid)s into solution from Neves Corvo waste rock.

In comparison to all halophilic organisms screened for bioleaching, *T. thiocyanaticus* seemed to be the least capable of bioleaching. This is not surprising, as bacterial growth and activity were quite low in the presence of NC_01. Sulphur oxidation was limited, with little variation in sulphate concentration and an increase in solution pH, throughout the experiment. This could be due to its low tolerance to the metal(loid)s contents of NC_01. The remaining three organisms (*T. cyclica*, *T. electrotropha* and *T. pacifica*) displayed an impressive level of tolerance to the metal(loid) content of NC_01. This was indicated by their ability to oxidize sulphur in the presence of NC_01, assessed by the increased sulphate concentration and decreased solution pH. Though *T. cyclica* mobilised substantial amounts of As (46.2%), K (31.2%), Sb (38.7%) and Ag (45.3%), the two *Thioclava* species were the most promising organisms for bioleaching in this study. This is because they both mobilised a wider range of metal(loid)s into solution, as between 8–30% of the metal(loid)s analysed were recovered by *T. electrotropha* and *T. pacifica*. In addition, the two *Thioclava* species were the only organisms that seemed to utilize the sulphides present in NC_01. The amount of sulphate produced in the bioleaching media with the two *Thioclava* species was ~43% higher than that produced by the bacterial pure cultures without NC_01 (data not shown), using the same nutrient medium and for the same duration of time. However, the pure bacterial cultures (without NC_01) of the other organisms (*T. cyclica* and *T. thiocyanaticus*), produced comparable or higher (in the case of *T. thiocyanaticus*) amounts of sulphate, in comparison to their bioleaching assays. This suggests that *T. cyclica* and *T. thiocyanaticus* may have only utilized sulphur compounds present in their nutrient media, and not sulphur in the waste rock. The toxic effect of NC_01 contents on *T. thiocyanaticus* reduced its ability to grow and oxidise sulphur, hence the lower sulphate concentrations, compared to its pure cultures where growth and the ability to oxidise sulphur were not hindered.

The rapid changes in Fe concentrations (Figure 1k) of the bioleaching experiment with *T. pacifica* are not unusual and have been recorded by several studies [29,30]. As Fe is solubilized from the material, it is also rapidly consumed in the synthesis of intermediate and secondary minerals, such as schwertmannite or jarosite. At any point in time, the concentration of dissolved Fe detected is the balance between mobilized Fe and consumed Fe. Therefore, higher Fe concentrations indicate that the mobilization of Fe dominated at that stage. While a lower Fe concentration indicates that the consumption of Fe dominated at that stage. To confirm that the *Thioclava* spp. were indeed promising for bioleaching, the bioleaching experiments with *T. electrotropha* and *T. pacifica* were repeated and similar results were obtained (Tables S8 and S9). For example, the maximum Cu recovery by *T. electrotropha* and *T. pacifica* in the first experiments were 8.7% and 8.1%, respectively. While in the second experiment, the maximum Cu recovery by *T. electrotropha* and *T. pacifica* were 8.5% and 13.1%, respectively.

The three different bioleaching assays (1SB, 2SB and SMB) helped to assess if the contents of NC_01 were toxic to the growth and bioleaching capacities of the organisms. In 2SB, biomass growth was initially separated from metal(loid) leaching by attaining the maximum cell density in the bioleaching medium, before exposing the microbe to the metal(loid)-containing solid material. This strategy could reduce the toxic effects of the solid material on the microbe, as a high biomass concentration increases tolerance to metal toxicity [31,32]. In this study, 2SB was not necessary for the bioleaching experiments, as no major difference was observed in the growth, activities or metal(loid) recovery levels of 1SB and 2SB for all bacteria. The 2SB was, however, very important for the microwave-assisted bioleaching experiment. After microwave roasting, NC_01 became quite toxic to the growth and activity of *T. electrotropha*. This was evident by the lack of sulphur oxidation and hence, little or no production of sulphuric acid by the microbe in 1SB. This was in addition to the consistent increase in pH and low metal(loid) recovery during the 1SB bioleaching experiments.

Microwave roasting generally transforms sulphide minerals into their more soluble sulphate and/or oxide forms, thereby increasing the extractability of target metals. After microwave roasting in this study, a high concentration of metal(loid)s in NC_01 became highly soluble, and therefore, more toxic to the growth of the bacteria. This was why little or no bacterial growth and activity were detected in 1SB. On the other hand, allowing the bacteria to grow up to the exponential stage before adding the microwave-roasted NC_01, increased the microbe’s tolerance to metal toxicity in 2SB. Subsequently, the oxidation of thiosulphate in the nutrient medium by the bacteria led to an increased sulphate concentration, reduced pH and finally, increased metal(loid) dissolution. The XRD results showed that most of the pyrite present in the waste rock was oxidised to hematite (Fe_2_O_3_) after microwave roasting at 400, 500 and 600 °C. The amount of pyrite decreased as the roasting temperatures increased, while the amount of hematite increased as the roasting temperatures increased (Table A12). Tailings originating from the same mine as the waste rock (NC_01) in this study were previously roasted in a microwave and characterised [33]. The results revealed that most of the pyrite present in the tailings was directly oxidised to magnetite (Fe_3_O_4_) and finally to hematite (Fe_2_O_3_) after microwave roasting at 400–600 °C. There was also a small amount of ferric sulphate (Fe_2_(SO4)_3_), which decreased as the roasting temperatures increased. The authors concluded that the strong microwave absorption by pyrite and other sulphide minerals promoted the rapid and selective heating of these mineral phases, quickly decomposing the formed sulphates and finally resulting in a fast sulphide-to-oxide mineral transition [33]. Microwave roasting is a fast, non-contact, selective and energy-efficient heating technology [34], and it successfully improved the bioleaching rates of Pb, Ag and In by *T. electrotropha* in this study.

Though most sulphides were converted to their sulphate and oxide forms by microwave roasting, XRD and XRF (Table A13) revealed that pyrite and sulphur still remained in the roasted samples. Therefore, the improved bioleaching of elements from the microwave-roasted samples may have been as a result of sulphide mineral oxidation, to an extent. In addition, the bacteria produced sulphuric acid which helped to leach elements (Pb, Ag and In) that could not be leached by water (the abiotic control). The bacteria may have also synthesized some metabolites such as amino acids that could bind these elements. For example, methionine has been shown to bind strongly to Ag [35]. The thiosulphate present in the nutrient medium may have also been responsible for the leaching of Ag, as thiosulphate can effectively leach precious metals [36]. Complexation by organic acids can be ruled out, as no organic acids were detected in *T. electrotropha* cultures by high-performance liquid chromatography (data not shown).

The bioleaching efficiencies for metals with economic importance (e.g., Zn and Co) by the four organisms in this study were quite low. Especially when compared to the conventional (non-halotolerant) bioleaching acidophilic consortium, which recovered over 70% of the total Zn, Co, and In contents of NC_01 and 32–55% Mn and Cu, within 21 days [37]. The bioleaching rates of the two *Thioclav*a spp. may be improved when used in combination with compatible halophilic Fe-oxidising bacteria such as *Mariprofundus ferrooxydans PV-1*, a neutrophilic bacterium isolated from Fe-rich microbial mats associated with hydrothermal venting at a submarine volcano, Loihi Seamount [38]. The high amount of acid-insoluble sulphide minerals (e.g., pyrite) in NC_01 necessitates the presence of Fe-oxidising organisms in the bioleaching medium, for the oxidation of Fe(II) to Fe(III). This is because Fe(III) is a more potent oxidant of acid-insoluble sulphide minerals than protons (H^+^) [39], as illustrated in the thiosulphate pathway of sulphide mineral oxidation (Equations (1)–(3)) [40]. Nevertheless, the two *Thioclav*a spp. may be more effective in bioleaching of materials with more acid-soluble minerals, such as galena or sphalerite, as the metal–sulphur bonds can be disrupted by protons [41].

FeS_2_ + 6 Fe^3+^ + 3 H_2_O → S_2_O_3_^2−^ + 7 Fe^2+^ + 6 H^+^ (1)

2 Fe^2+^ + 2 H^+^ + 0.5 O_2_ → 2 Fe^3+^ + H_2_O (2)

S_2_O_3_^2−^ + 2 O_2_ + H_2_O → 2 SO_4_^2−^ + 2 H^+^ (3)

The ability of the present halophilic sulphur-oxidising bacteria to oxidise mineral sulphides and mobilise metal(loid)s from mine waste into solution should raise environmental concerns. This is because, similar neutrophilic/alkaliphilic organisms with the same functions (e.g., sulphur oxidation) have been shown to be present in sulphidic mine waste dumps. For example, a high abundance of neutrophilic (halophilic) sulphur-oxidising organisms from the *Halothiobacillus* and *Thiomicrospira* genera have recently been isolated from the Neves Corvo ore and fresh tailings samples [42]. These organisms (including other types of bacteria isolated) were able to mobilise As (96%), Cd (58%), Cu (24%) and Zn (56%) from tailings obtained from an abandoned tailings dump in Barroca Grande [42]. Little is known about the activities and roles of these neutrophilic/alkaliphilic sulphur-oxidising organisms in the development of acid mine drainage (AMD) in mining habitats. Yet, they are very relevant in the biogeochemistry of sulphidic ores and mine wastes. Some studies suggest that such neutrophilic sulphur-oxidising bacteria are responsible for the first series of oxidation processes that reduce the pH of fresh sulphidic mine habitats from alkaline (or circumneutral) to acidic values, before the prevalence of acidophilic iron- and/or sulphur-oxidizing prokaryotes accelerating the oxidation process further at pH <3 or in AMD environments [43,44]. The bioleaching experiments presented here support these observations, indicating that the acidification of sulphidic mine dumps (e.g., tailings and waste rock) and the solubilization of toxic metal(loid)s are first-order effects of the actions of neutrophilic (halophilic) sulphur-oxidizing organisms such as the ones used in this study. Neutrophilic sulphur-oxidizing organisms are as important as acidophilic iron- and/or sulphur-oxidizing organisms, as they are all strong contributors to the long-term environmental threat of acid generation and metal(loid) solubilization in sulphidic mine habitats.

The higher sulphate productions of *T. electrotropha* and *T. pacifica* in the presence of NC_01 during bioleaching, compared to the pure cultures under the same conditions, suggest that the two organisms were able to utilise or oxidise the mineral sulphides in NC_01. Therefore, a decrease in the concentrations of sulphide minerals in NC_01 was expected after bioleaching. However, no major consistent changes were detected in the mineral compositions of NC_01 before or after bioleaching by the MLA. This could be due to the very low concentrations of non-pyrite and acid-soluble sulphide minerals in NC_01 (≤0.6 wt% each) and also the limited accuracy level of the MLA technique for low concentrations of minerals. This suggests that, though the two *Thioclava* spp. might have changed the mineralogy of NC_01 during bioleaching, these changes occurred at minuscule levels due to the very low concentrations of several minerals and were therefore too little to be detected by the MLA with a high degree of confidence. In addition, the Neves Corvo waste rock samples were quite heterogenous, which could have contributed to the variations or inconsistent data obtained for some minerals.

## 5. Conclusions

This study demonstrated the ability of four neutrophilic and alkaliphilic sulphur-oxidising bacteria to mobilise metal(loid)s into solution from the Neves Corvo sulphidic mine waste rock, in the presence of chloride ions. The low bioleaching efficiencies of the organisms indicate limited industrial potential. However, the applied and suggested optimization processes for the two promising organisms (*T. electrotropha* and *T. pacifica*) may present significant benefits, such as saline water bioleaching and the biooxidation of sulphidic ores and wastes, especially in regions with fresh water scarcity. Further studies will incorporate metal(loid) recovery from leachates using selective techniques such as sulphide precipitation and solvent extraction. The methods described in this study can be applied in other studies, as the search for competent halophilic bioleaching organisms continues. We should, however, note that the new halophilic bioleaching organisms will have to function as microbial consortia rather than as individuals, which is the key to the success of conventional bioleaching organisms.

## Figures and Tables

**Table 1:** Chemical composition of the Neves Corvo waste rock (NC_01).

Elements (mg/kg)	Cu	Pb	Zn	Fe	S	Co	As	Cd	K	Mn	Ag	In	Sb
NC_01	1800	800	3600	104,500	56,100	85	800	10	14,000	760	6	4	45

## Data Availability

The data presented in this study are available on request from the corresponding author. The data are not publicly available yet because they are yet to be placed in an online repository.

## Supplementary Materials

The following supporting information can be downloaded at: https://www.mdpi.com/article/10.3390/microorganisms11010222/s1.

## Appendix A

**Table A1:** Percentages of the metal(loid)s extracted from the Neves Corvo waste rock by *T. cyclica* via one-step bioleaching (1SB), two-step bioleaching (2SB), spent medium bioleaching (SMB), and the abiotic control (AC). Values are the average of duplicate flasks ± standard deviations.

Metal(loid)s (%)	Days	1SB	2SB	SMB	AC
Cu	Day-7	12.5 ± 0.1	1.3 ± 0	1.6 ± 0	0 ± 0
	Day-14	9.2 ± 0.1	3.1 ± 0.1	1.6 ± 0	0 ± 0
	Day-21	7.8 ± 0	3.8 ± 0.1	1.2 ± 0	0 ± 0
	Day-28	6 ± 0.1	4 ± 0	1 ± 0.1	0 ± 0
Pb	Day-7	4 ± 0.6	5.2 ± 0	5 ± 0.5	0 ± 0
	Day-14	2 ± 0.1	1.7 ± 0.1	1.5 ± 0.2	0 ± 0
	Day-21	1.3 ± 0.1	0.7 ± 0	0.7 ± 0	0 ± 0
	Day-28	0.6 ± 0	0.4 ± 0	0.3 ± 0.2	0 ± 0
Zn	Day-7	1.3 ± 0.5	0.1 ± 0	0.1 ± 0	0.2 ± 0
	Day-14	1.1 ± 0	0.3 ± 0	0.1 ± 0	0.4 ± 0.2
	Day-21	1.2 ± 0	0.4 ± 0	0.2 ± 0	0.4 ± 0.1
	Day-28	0.8 ± 0.1	0.5 ± 0	0.1 ± 0	0.8 ± 0.5
Co	Day-7	0 ± 0.2	2.4 ± 0.1	2.8 ± 0.1	1.7 ± 0.2
	Day-14	0.7 ± 0	3.4 ± 0.2	5.1 ± 0.3	2.4 ± 0.8
	Day-21	1.4 ± 0.1	4.8 ± 0.3	6.9 ± 0.4	2.4 ± 0.6
	Day-28	2.1 ± 0.1	6.2 ± 0.2	6 ± 0.2	3.6 ± 1.7
As	Day-7	41 ± 2.6	16.6 ± 0.4	28.3 ± 0.2	0 ± 0
	Day-14	44.6 ± 0.7	33.1 ± 0.4	36.1 ± 1.3	0.1 ± 0
	Day-21	46.2 ± 1.8	40 ± 0.2	36.1 ± 1.3	0 ± 0
	Day-28	44.2 ± 2.8	42.4 ± 0.2	33.4 ± 0	0 ± 0
Cd	Day-7	4.1 ± 0.3	0.6 ± 0.1	0.9 ± 0	1.4 ± 0.2
	Day-14	3.1 ± 0.2	1 ± 0.1	0.7 ± 0.1	1.9 ± 0.6
	Day-21	3.1 ± 0.2	1.1 ± 0	0.7 ± 0.1	2 ± 0.5
	Day-28	2.3 ± 0.3	1.1 ± 0.1	0.5 ± 0.1	2.7 ± 0.9
K	Day-7	31.2 ± 1.1	28.1 ± 0	6.1 ± 1.2	1.8 ± 0.1
	Day-14	12.5 ± 0.9	9.7 ± 0.9	0 ± 0	1.6 ± 0
	Day-21	16.7 ± 0.9	11.1 ± 1.3	49.5 ± 4.7	2 ± 0.3
	Day-28	9.4 ± 0.8	6 ± 0.9	17.4 ± 0.6	1.8 ± 0.1
Sb	Day-7	25 ± 0.5	6.1 ± 0.4	11.4 ± 0.4	0.4 ± 0
	Day-14	29.8 ± 0.9	13.6 ± 0.6	15.6 ± 0.1	0.3 ± 0
	Day-21	34.6 ± 0.7	19.3 ± 1	21 ± 0.6	0.3 ± 0
	Day-28	38.7 ± 0.5	24.2 ± 1.2	20 ± 0	0.2 ± 0
Ag	Day-7	44.2 ± 0.5	0 ± 0	1.3 ± 0.5	0 ± 0
	Day-14	45.3 ± 0.5	4.5 ± 0.3	0 ± 0	0 ± 0
	Day-21	33.9 ± 1.1	17.9 ± 0.8	0.7 ± 0	3.8 ± 0.5
	Day-28	23.7 ± 2.2	17.8 ± 0.7	0 ± 0	2.4 ± 1
Mn	Day-7	0.1 ± 0	0 ± 0	0 ± 0	0.7 ± 0.1
	Day-14	0 ± 0	0 ± 0	0 ± 0	8.5 ± 1
	Day-21	0 ± 0	0 ± 0	0 ± 0	9.1 ± 1.2
	Day-28	0 ± 0	0 ± 0	0 ± 0	10.1 ± 1.9

**Table A2:** Percentages of the metal(loid)s extracted from the Neves Corvo waste rock by *T. thiocyanaticus* via one-step bioleaching (1SB), two-step bioleaching (2SB), spent medium bioleaching (SMB), and the abiotic control (AC). Values are the average of duplicate flasks ± standard deviations.

Metal(loid)s (%)	Days	1SB	2SB	SMB	AC
Cu	Day-7	7.3 ± 0.1	1 ± 0	1 ± 0.1	0 ± 0
	Day-14	2.4 ± 0	1.4 ± 0.1	0.9 ± 0.1	0 ± 0
	Day-21	1.9 ± 0.1	2 ± 0.1	0.8 ± 0.1	0 ± 0
	Day-28	1.7 ± 0	2.1 ± 0.4	0.9 ± 0.1	0 ± 0
Pb	Day-7	0.3 ± 0	0.3 ± 0	0.4 ± 0	0 ± 0
	Day-14	0.1 ± 0	0.1 ± 0	0.1 ± 0	0 ± 0
	Day-21	0.1 ± 0	0.1 ± 0	0 ± 0	0 ± 0
	Day-28	0.1 ± 0	0.1 ± 0	0 ± 0	0 ± 0
Zn	Day-7	0.1 ± 0	0 ± 0	0.1 ± 0	0.2 ± 0
	Day-14	0 ± 0	0.1 ± 0	0 ± 0	0.2 ± 0
	Day-21	0.1 ± 0	0 ± 0	0 ± 0	0.2 ± 0
	Day-28	0.1 ± 0	0 ± 0	0 ± 0	0.2 ± 0.1
Co	Day-7	1.3 ± 0	1.3 ± 0	0.8 ± 0.1	1.2 ± 0.1
	Day-14	1.1 ± 0.1	0.3 ± 0.2	0 ± 0	1.2 ± 0.4
	Day-21	1.1 ± 0	0 ± 0	0.6 ± 0.2	0.9 ± 0.2
	Day-28	1 ± 0.2	0 ± 0	0.6 ± 0.2	1.2 ± 0.6
As	Day-7	0.7 ± 0.1	1.1 ± 0	1.1 ± 0	0 ± 0
	Day-14	2.7 ± 0.1	1.4 ± 0	1.6 ± 0	0 ± 0
	Day-21	2.6 ± 0.1	1.6 ± 0	1.6 ± 0	0 ± 0
	Day-28	2.7 ± 0.2	1.4 ± 0.3	1.7 ± 0	0 ± 0
Cd	Day-7	3.3 ± 0.4	2.7 ± 0	3.2 ± 0.5	1 ± 0.1
	Day-14	1.3 ± 0.1	0.8 ± 0.1	2.6 ± 0.9	1 ± 0.4
	Day-21	1.7 ± 0	0.7 ± 0.2	2.1 ± 0.7	0.9 ± 0.2
	Day-28	1.3 ± 0.2	0.7 ± 0.3	1.8 ± 0.7	1 ± 0.3
K	Day-7	5.2 ± 0.3	4.4 ± 0.8	0 ± 0	1.5 ± 0.1
	Day-14	7.2 ± 0.4	5.4 ± 0.8	50.7 ± 6.9	1.6 ± 0
	Day-21	5.9 ± 1.6	6.3 ± 0.1	11.8 ± 4	1.7 ± 0
	Day-28	0 ± 0	0 ± 0	20.6 ± 4.7	1.3 ± 0
Sb	Day-7	25 ± 0.5	6.1 ± 0.4	11.4 ± 0.4	0.4 ± 0
	Day-14	29.8 ± 0.9	13.6 ± 0.6	15.6 ± 0.1	0.3 ± 0
	Day-21	34.6 ± 0.7	19.3 ± 1	4.8 ± 0.5	0.3 ± 0
	Day-28	38.7 ± 0.5	24.2 ± 1.2	4.8 ± 0.4	0.2 ± 0
Ag	Day-7	14.2 ± 1.5	0 ± 0	0.1 ± 0	0 ± 0
	Day-14	31.8 ± 0.6	20.1 ± 1.7	1.5 ± 0.8	3.5 ± 0.3
	Day-21	15 ± 1.6	11.2 ± 0.5	1.6 ± 1	2.3 ± 1.1
	Day-28	22.7 ± 0.4	13.6 ± 2.9	1.6 ± 1	1.1 ± 0.3
Mn	Day-7	0 ± 0	0 ± 0	0.3 ± 0	6.4 ± 0.4
	Day-14	0 ± 0	0 ± 0	0.4 ± 0.1	6.6 ± 0.7
	Day-21	0 ± 0	0 ± 0	0 ± 0	6.2 ± 1
	Day-28	0 ± 0	0 ± 0	0.3 ± 0.1	6.7 ± 1.5

**Table A3:** Percentages of the metal(loid)s extracted from the Neves Corvo waste rock by *T. electrotropha* via one-step bioleaching (1SB), two-step bioleaching (2SB), spent medium bioleaching (SMB), and the abiotic control (AC). Values are the average of duplicate flasks ± standard deviations.

Metal(loid)s (%)	Days	1SB	2SB	SMB	AC
Cu	Day-7	2.2 ± 0.2	1.7 ± 0.3	1.8 ± 0.3	0 ± 0
	Day-14	4.9 ± 0.3	3.9 ± 0.3	2.2 ± 0.2	0 ± 0
	Day-21	6.7 ± 0.4	4.5 ± 0.4	4.4 ± 0.8	0 ± 0
	Day-28	8.7 ± 0.7	7 ± 1.6	7.3 ± 1	0 ± 0
Pb	Day-7	0.5 ± 0.1	3.3 ± 1.3	0 ± 0	0 ± 0
	Day-14	7.5 ± 0.2	13.7 ± 0.8	0 ± 0	0 ± 0
	Day-21	9.5 ± 0.1	9.9 ± 0.1	10 ± 2.6	0 ± 0
	Day-28	9.8 ± 0.4	9.6 ± 0	12.4 ± 0.7	0 ± 0
Zn	Day-7	5 ± 0.3	7.3 ± 0.4	0.1 ± 0	0.2 ± 0.1
	Day-14	8.4 ± 0.2	8.8 ± 0.1	0 ± 0	0.3 ± 0.1
	Day-21	10.3 ± 0.1	9.3 ± 0.2	8.9 ± 0.9	0.3 ± 0.2
	Day-28	12.3 ± 0.4	10.3 ± 1	12.8 ± 2.1	0.7 ± 0.1
Co	Day-7	22.5 ± 0.1	22.7 ± 0.2	9.6 ± 0.6	1.5 ± 0.2
	Day-14	24.2 ± 0.2	25.1 ± 0.5	7.5 ± 1	1.8 ± 0.3
	Day-21	25 ± 0.7	24.1 ± 1.4	21 ± 2.4	1.5 ± 0.5
	Day-28	24.9 ± 0.8	23.5 ± 1.5	24.4 ± 1.9	1.9 ± 0.3
As	Day-7	0.5 ± 0.1	0.6 ± 0.1	0.3 ± 0	0 ± 0
	Day-14	0.9 ± 0	0.7 ± 0	0.4 ± 0.1	0 ± 0
	Day-21	1.8 ± 0.1	3.3 ± 0.6	0.7 ± 0.1	0 ± 0
	Day-28	3.3 ± 0.2	4.8 ± 0.7	2.1 ± 1.3	0 ± 0
Cd	Day-7	13.3 ± 0.4	13.2 ± 0.3	4.7 ± 0.8	1 ± 0.2
	Day-14	14.1 ± 0.5	13.2 ± 0.4	2.1 ± 0.3	1.3 ± 0.4
	Day-21	14.1 ± 0.8	12.9 ± 0.7	12.6 ± 0.9	1.2 ± 0.5
	Day-28	16 ± 1.2	13.7 ± 1.8	16.9 ± 2.5	2.2 ± 0.4
K	Day-7	0 ± 0	0 ± 0	6 ± 0.8	2 ± 0
	Day-14	0 ± 0	0 ± 0	0 ± 0	2 ± 0
	Day-21	9.2 ± 0.4	7.7 ± 0.4	0 ± 0	2.1 ± 0.3
	Day-28	0 ± 0	0 ± 0	21 ± 10.1	2.1 ± 0
Sb	Day-7	1.2 ± 0.1	0.8 ± 0.1	1.2 ± 0	0.7 ± 0
	Day-14	1.6 ± 0.1	0.9 ± 0	2.2 ± 0.1	0.7 ± 0
	Day-21	1.6 ± 0	1.3 ± 0.2	1.5 ± 0.4	0.9 ± 0
	Day-28	2.1 ± 0.3	1.8 ± 0.5	1.6 ± 0.3	2.1 ± 0.9
Ag	Day-7	0 ± 0	0.8 ± 0.5	0 ± 0	0 ± 0
	Day-14	3.3 ± 0.2	4.9 ± 0.5	0.9 ± 0.1	0 ± 0
	Day-21	3.1 ± 0.3	5.3 ± 1	4.7 ± 2.1	0 ± 0
	Day-28	4 ± 0.4	3.6 ± 0.3	5.2 ± 1.2	0 ± 0
Mn	Day-7	13.2 ± 0.1	12.6 ± 0	7.7 ± 0.3	5.5 ± 0.3
	Day-14	13.4 ± 0	14.3 ± 0.2	3.4 ± 0.5	6.2 ± 0.5
	Day-21	13.6 ± 0.6	13.7 ± 0.6	11.8 ± 2	6.1 ± 1.5
	Day-28	12.8 ± 0.2	12.7 ± 0.4	13.9 ± 0.7	7 ± 0.8

**Table A4:** Percentages of the metal(loid)s extracted from the Neves Corvo waste rock by *T. pacifica* via one-step bioleaching (1SB), two-step bioleaching (2SB), spent medium bioleaching (SMB), and the abiotic control (AC). Values are the average of duplicate flasks ± standard deviations.

Metal(loid)s (%)	Days	1SB	2SB	SMB	AC
Cu	Day-7	2.3 ± 0.1	1.3 ± 0.2	2 ± 0	0 ± 0
	Day-14	5 ± 0.8	1.9 ± 0.2	2.4 ± 0.1	0 ± 0
	Day-21	6.5 ± 0.8	3.7 ± 0.4	4.3 ± 0	0 ± 0
	Day-28	8.1 ± 0.5	5 ± 1.1	7.3 ± 0.2	0 ± 0
Pb	Day-7	0.3 ± 0.1	4.5 ± 0.1	0.3 ± 0	0 ± 0
	Day-14	8.9 ± 1.7	0.3 ± 0	0.3 ± 0	0 ± 0
	Day-21	9.9 ± 0.9	8.7 ± 1.1	12.8 ± 0.2	0 ± 0
	Day-28	9.9 ± 0.8	10.1 ± 1.7	13 ± 0.4	0 ± 0
Zn	Day-7	2.2 ± 0.8	6.7 ± 0.1	2.2 ± 0.1	0.2 ± 0.1
	Day-14	9 ± 1	0.7 ± 0.2	0.7 ± 0.2	0.3 ± 0.1
	Day-21	10.9 ± 1.9	8.1 ± 0	9.2 ± 0	0.3 ± 0.2
	Day-28	12.1 ± 1.3	9.4 ± 0.8	12.6 ± 1	0.7 ± 0.1
Co	Day-7	18.8 ± 1.4	21.8 ± 0.2	20.9 ± 0.3	1.5 ± 0.2
	Day-14	25.9 ± 0.2	13.7 ± 0.7	13.3 ± 1.2	1.8 ± 0.3
	Day-21	26.7 ± 1	23.7 ± 0.5	23.8 ± 0.3	1.5 ± 0.5
	Day-28	26 ± 0	24.1 ± 0.3	26.7 ± 1	1.9 ± 0.3
As	Day-7	0.8 ± 0	0.7 ± 0	0.3 ± 0	0 ± 0
	Day-14	1.1 ± 0.2	0.5 ± 0	0.4 ± 0	0 ± 0
	Day-21	1.6 ± 0.5	0.6 ± 0.1	0.7 ± 0	0 ± 0
	Day-28	2.7 ± 1	1.7 ± 0.2	2.5 ± 0.5	0 ± 0
Cd	Day-7	12.3 ± 0.6	13.5 ± 0.1	10.7 ± 0.1	1 ± 0.2
	Day-14	15.1 ± 0.8	7.3 ± 0.9	7 ± 0.8	1.3 ± 0.4
	Day-21	15 ± 1.1	12.1 ± 0	12.8 ± 0.3	1.2 ± 0.5
	Day-28	16.8 ± 0.9	13.1 ± 0.4	16.5 ± 1.4	2.2 ± 0.4
K	Day-7	0 ± 0	0 ± 0	5.3 ± 0.7	2 ± 0
	Day-14	0 ± 0	0 ± 0	0 ± 0	2 ± 0
	Day-21	7.8 ± 0.6	6.8 ± 1.2	0 ± 0	2.1 ± 0.3
	Day-28	0 ± 0	0 ± 0	9.3 ± 2.1	2.1 ± 0
Sb	Day-7	1.6 ± 0.2	0.9 ± 0	1.1 ± 0	0.7 ± 0
	Day-14	1.5 ± 0.1	1.2 ± 0	2 ± 0.1	0.7 ± 0
	Day-21	1.6 ± 0.1	0.9 ± 0.1	1.1 ± 0	0.9 ± 0
	Day-28	2.2 ± 0	1.1 ± 0.2	1.6 ± 0.3	2.1 ± 0.9
Ag	Day-7	0 ± 0	0 ± 0	2.7 ± 0.6	0 ± 0
	Day-14	2.1 ± 0	0 ± 0	0 ± 0	0 ± 0
	Day-21	3.6 ± 0.3	3.1 ± 0	5 ± 1.6	0 ± 0
	Day-28	4.4 ± 0.7	2.9 ± 0.2	3.8 ± 0.1	0 ± 0
Mn	Day-7	9.8 ± 1.4	11.8 ± 0.3	11.5 ± 0.1	5.5 ± 0.3
	Day-14	11.7 ± 0.9	7.1 ± 0.5	7.2 ± 0.8	6.2 ± 0.5
	Day-21	12.1 ± 0.4	11.5 ± 0.4	12.2 ± 0.5	6.1 ± 1.5
	Day-28	11.3 ± 0.6	11.7 ± 0.4	13.3 ± 0.4	7 ± 0.8

**Table A5:** Percentages of the metal(loid)s extracted from the Neves Corvo waste rock by the *Thioclava* consortium via one-step bioleaching (1SB), two-step bioleaching (2SB), and spent medium bioleaching (SMB). Values are the average of duplicate flasks ± standard deviations.

Metal(loid)s (%)	Days	1SB	2SB	SMB
Cu	Day-7	3.2 ± 0.1	1.8 ± 0.5	1.7 ± 0.7
	Day-14	4.9 ± 0	2.1 ± 0.1	2.5 ± 0.2
	Day-21	7 ± 0	3.6 ± 0.1	4.8 ± 0.8
	Day-28	9.2 ± 0.7	5.3 ± 0.5	9 ± 1.5
Pb	Day-7	0.9 ± 0	0.7 ± 0	0.1 ± 0
	Day-14	2.2 ± 1.2	0 ± 0	3.5 ± 4.9
	Day-21	10.4 ± 0.2	9.8 ± 0.5	12.9 ± 1.5
	Day-28	10.7 ± 0.8	13.4 ± 1.8	18.6 ± 7.8
Zn	Day-7	7 ± 1.2	7.2 ± 2	7.8 ± 1
	Day-14	7.2 ± 0.8	7.3 ± 0	8 ± 0
	Day-21	10.1 ± 0.9	8.7 ± 0.3	9.9 ± 0.6
	Day-28	12.9 ± 1.5	10.2 ± 0.1	13.7 ± 2.1
Co	Day-7	24.7 ± 0.5	23.7 ± 1.9	24.2 ± 1.9
	Day-14	22 ± 0.9	6.8 ± 1.3	25.3 ± 0
	Day-21	24.1 ± 0.9	21.7 ± 0.5	26.2 ± 1.7
	Day-28	26 ± 1.3	23.4 ± 0.9	29.6 ± 3.5
As	Day-7	2 ± 0.2	0.5 ± 0.1	0.8 ± 0.5
	Day-14	1.3 ± 0	0.4 ± 0.1	0.6 ± 0.4
	Day-21	1.7 ± 0.2	0.6 ± 0.2	3.9 ± 0.5
	Day-28	2.7 ± 0.2	2 ± 0.5	2.4 ± 0.9
Cd	Day-7	12.1 ± 0.2	11.9 ± 0.4	10.8 ± 0.3
	Day-14	12.5 ± 0.3	2.6 ± 0.5	13 ± 0
	Day-21	14.4 ± 0.9	12.5 ± 0	13.9 ± 0.6
	Day-28	17.2 ± 2.3	14 ± 0.4	20.2 ± 2.9
K	Day-7	3.6 ± 1.1	3.3 ± 0.1	0 ± 0
	Day-14	0 ± 0	0 ± 0	7.6 ± 1.1
	Day-21	0 ± 0	0 ± 0	16.9 ± 12.4
	Day-28	6.2 ± 0.5	6.2 ± 1	19.6 ± 1.3
Sb	Day-7	1.3 ± 0.1	1 ± 0.3	1.7 ± 0.1
	Day-14	2 ± 0.2	1.9 ± 0.4	2.5 ± 0.5
	Day-21	2 ± 0.2	1.3 ± 0.1	1.9 ± 0
	Day-28	2.5 ± 0.4	1.3 ± 0.1	3.2 ± 0.1
Ag	Day-7	0 ± 0	1.1 ± 0.4	1 ± 0.1
	Day-14	3 ± 0.4	0 ± 0	1.8 ± 0
	Day-21	3.9 ± 0.5	5.5 ± 1	4.8 ± 1.8
	Day-28	3.7 ± 0.7	6.1 ± 0	3.1 ± 0.5
Mn	Day-7	14.8 ± 0.2	13.1 ± 0.8	13.6 ± 0
	Day-14	12.9 ± 0.9	4.3 ± 0.5	14.1 ± 0
	Day-21	13.6 ± 1.1	12.3 ± 0.2	14.6 ± 0.8
	Day-28	14.1 ± 1.2	13.9 ± 0	14.7 ± 0.6

**Table A6:** Percentages of the metal(loid)s extracted from the Neves Corvo waste rock (NC_01) via one-step bioleaching and two-step bioleaching by *T. electrotropha* (E-1SB & E-2SB), as well as one-step bioleaching and two-step bioleaching by *T. pacifica* (P-1SB & P-2SB). Results of weekly serial addition of NC_01 and partial nutrient replacement. Values are the average of duplicate flasks ± standard deviations.

Metal(loid)s (%)	Days	Electrotropha 1SB	Electrotropha 2SB	Pacifica 1SB	Pacifica 2SB
Cu	Day-7	931.1 ± 2.8	3.8 ± 1.3	4.9 ± 0.1	2.4 ± 0.3
	Day-14	1319.1 ± 84.9	2.4 ± 0.2	3.9 ± 1.5	2.3 ± 0.2
	Day-21	4479.1 ± 466.7	2.4 ± 0.3	2.7 ± 0	3.2 ± 1.3
	Day-28	5554.1 ± 332.3	3.9 ± 0.1	3.9 ± 0	3.1 ± 0
	Day-35	4884.1 ± 912.2	4.5 ± 0.3	4.9 ± 0	8.1 ± 0.6
	Day-42	4379.1 ± 466.7	4 ± 0.7	5.8 ± 1.1	6.3 ± 0.3
Pb	Day-7	684.3 ± 166.9	20.7 ± 7.8	2.3 ± 0	2.4 ± 0
	Day-14	128.3 ± 181	0 ± 0	3.3 ± 3.4	0.5 ± 0.1
	Day-21	3962.3 ± 898	7.6 ± 0.2	1.2 ± 1	1.1 ± 0.2
	Day-28	2577.3 ± 183.8	5.5 ± 0.6	4.9 ± 1.5	2.8 ± 2.7
	Day-35	1472.3 ± 374.8	4 ± 0.4	3.6 ± 0.8	4 ± 1.2
	Day-42	1087.3 ± 84.9	3.2 ± 0.8	2.5 ± 0.6	2.8 ± 0.8
Zn	Day-7	3598.7 ± 0	10.2 ± 0.3	7.2 ± 0.3	7.4 ± 2.2
	Day-14	4298.7 ± 0	0 ± 0	0.4 ± 0	0.2 ± 0
	Day-21	17,108.7 ± 0	8.1 ± 0.1	9.4 ± 0.1	6.9 ± 1.4
	Day-28	16,758.7 ± 70.7	7.3 ± 0.1	10.1 ± 2.4	10.4 ± 5.2
	Day-35	13,358.7 ± 2757.7	6.4 ± 0.4	8.5 ± 0.6	10.3 ± 0.9
	Day-42	11,358.7 ± 636.4	5.7 ± 0.7	7 ± 0.2	7 ± 0.4
Co	Day-7	263.5 ± 0.7	30.6 ± 0.8	30.1 ± 2.2	29.5 ± 3.5
	Day-14	332 ± 0	9.2 ± 1	6.9 ± 2.2	7.7 ± 2.2
	Day-21	881.5 ± 7.8	18.8 ± 0.4	21.2 ± 4.7	15.5 ± 0
	Day-28	690 ± 8.5	15.2 ± 0	16.8 ± 0.1	12.8 ± 3.5
	Day-35	465.5 ± 65.8	10.7 ± 0.3	13.2 ± 0.6	12.2 ± 0.8
	Day-42	319 ± 21.2	7.1 ± 0.9	9.3 ± 0.1	8.5 ± 0.3
As	Day-7	379 ± 9.9	3.4 ± 0.5	4.4 ± 0.2	2 ± 0.2
	Day-14	377.5 ± 37.5	1.5 ± 0.2	1 ± 0.4	1.2 ± 0.1
	Day-21	1516 ± 76.4	1.1 ± 0.1	1.1 ± 0.1	0.9 ± 0.3
	Day-28	2545 ± 162.6	5.2 ± 0.3	1.7 ± 0.8	1.1 ± 0.2
	Day-35	2730 ± 127.3	6.8 ± 0.1	6.5 ± 1.4	1.4 ± 0.3
	Day-42	2435 ± 558.6	5.6 ± 0.7	6.1 ± 0.1	2.2 ± 0.1
Cd	Day-7	11.1 ± 0.2	11.9 ± 0.4	10.9 ± 1.4	10.5 ± 0.9
	Day-14	7.6 ± 0	2 ± 0.2	4.6 ± 0	3.5 ± 0
	Day-21	55.5 ± 5.3	11.7 ± 0	15.4 ± 0	13.8 ± 1.9
	Day-28	50.1 ± 1.4	9.2 ± 0.4	12.1 ± 2.5	14.1 ± 3.7
	Day-35	38.7 ± 6.9	7.5 ± 0.6	9.4 ± 0.5	10.4 ± 0.2
	Day-42	36 ± 0.4	7.2 ± 0.8	8.4 ± 0.3	7.7 ± 0.6
K	Day-7	25,500 ± 7071.1	19 ± 0	15.6 ± 2.9	11.4 ± 1
	Day-14	0 ± 0	0 ± 0	0 ± 0	0 ± 0
	Day-21	0 ± 0	0 ± 0	0 ± 0	0 ± 0
	Day-28	51500 ± 0	7.2 ± 0.9	6.6 ± 0.1	6.1 ± 0.1
	Day-35	41500 ± 0	6.6 ± 0.2	6.2 ± 0.8	4.7 ± 0.3
	Day-42	123,500 ± 4242.6	16 ± 1.1	16 ± 0.5	13.9 ± 2.2
Sb	Day-7	11.4 ± 0.5	3.4 ± 0.5	2.6 ± 0	1.7 ± 0
	Day-14	41.8 ± 3.9	3.5 ± 0.4	2.1 ± 0.9	2.7 ± 0.4
	Day-21	48.8 ± 0	0.9 ± 0.1	2.1 ± 0	2.8 ± 0
	Day-28	36.9 ± 9.3	0.8 ± 0	1 ± 0	4.4 ± 0
	Day-35	36.5 ± 3.8	0.8 ± 0	1 ± 0	1.7 ± 0
	Day-42	39.1 ± 14.6	1.1 ± 0.1	1.2 ± 0	1.3 ± 0
Ag	Day-7	3.9 ± 0.4	3.9 ± 1	3.3 ± 0.5	0 ± 0
	Day-14	6 ± 0	3.9 ± 0	1.1 ± 0	0 ± 0
	Day-21	8.6 ± 1.8	3.6 ± 0.5	11.3 ± 0.5	0 ± 0
	Day-28	8.8 ± 1	3.2 ± 0.1	2.2 ± 0	4.1 ± 0
	Day-35	11.5 ± 2	3.8 ± 0.4	2.3 ± 0	11.6 ± 1.2
	Day-42	10.7 ± 2.3	5.5 ± 2.1	2.6 ± 0	8.4 ± 2
Mn	Day-7	1307.3 ± 14.1	18.4 ± 0.9	13.6 ± 0.1	12.6 ± 2.6
	Day-14	1755.8 ± 115.3	2.5 ± 0.7	12.6 ± 0	1.4 ± 0.5
	Day-21	4297.3 ± 113.1	10.9 ± 0.8	8.1 ± 0	7.7 ± 0.1
	Day-28	3127.3 ± 155.6	8.3 ± 0.3	7.8 ± 1.6	5.3 ± 1.1
	Day-35	1977.3 ± 282.8	5.4 ± 0.2	5.7 ± 1.1	5.1 ± 0.6
	Day-42	1262.3 ± 91.9	3.3 ± 0.3	3.9 ± 0.2	3.8 ± 0.6

**Table A7:** Concentrations and percentages of the metal(loid)s extracted from the Neves Corvo waste rock (NC_01) by the spent nutrient mediums of *T. electrotropha* and *T. pacifica* at high temperature and pressure. Values are the average of duplicate flasks ± standard deviations.

	Concentration (µg/L)		% Recovery	
Metal(loid)s	*T. electrotropha*	*T. pacifica*	*T. electrotropha*	*T. pacifica*
Cu	187.1 ± 8.5	246.6 ± 13.4	0.2 ± 0	0.3 ± 0
Pb	4287.3 ± 0	4362.3 ± 77.8	10.4 ± 0	10.6 ± 0.2
Zn	12,308.7 ± 0	11,708.7 ± 141.4	6.9 ± 0	6.6 ± 0.1
Co	1050 ± 0	997 ± 18.4	24.8 ± 0	23.5 ± 0.4
As	665.5 ± 48.8	556 ± 15.6	1.7 ± 0.1	1.5 ± 0
Cd	52.6 ± 0.2	53.2 ± 1.2	11.7 ± 0	11.8 ± 0.3
K	0.0	0.0	0.0	0.0
Sb	32.5 ± 0.4	33.7 ± 1.9	1.2 ± 0.1	0.7 ± 0
Ag	28.6 ± 2.6	32.4 ± 1	10 ± 0.9	11.4 ± 0.3
Mn	8407.3 ± 14.1	8992.3 ± 21.2	23.2 ± 0	24.8 ± 0.1
In	0.0	4.2 ± 0.1	0.0	2.3 ± 0.1
Fe	110,000 ± 0	142,500 ± 707.1	1.8 ± 0	2.3 ± 0

**Table A8:** pH and sulphate (SO_4_^2−^) concentration before and after bioleaching of the Neves Corvo waste rock (NC_01) by the spent mediums of *T. electrotropha* and *T. pacifica* at high temperature and pressure.

	*T. electrotropha*		*T. pacifica*	
	Before	After	Before	After
pH	2.7 ± 0.0	3.9 ± 0.0	2.8 ± 0.0	3.8 ± 0.0
SO_4_^2−^ concentration	137.6 ± 1.1	2210 ± 155.6	132.8 ± 0.6	1975 ± 63.6

**Table A9:** Percentages of the metal(loid)s extracted from the microwave-roasted (at 400, 500 and 600 °C) Neves Corvo waste rock (NC_01) by *T. electrotropha* via one-step bioleaching (1SB), two-step bioleaching (2SB) and the abiotic control (AC). Values are the average of duplicate flasks ± standard deviations.

Metal(loid)s (%)	Days	400 °C 1SB	400 °C 2SB	400 °C AC	500 °C 1SB	500 °C 2SB	500 °C AC	600 °C 1SB	600 °C 2SB	600 °C AC
Cu	Day-7	46.2 ± 0.1	49.3 ± 0.8	52 ± 0.5	59.2 ± 0.5	61.3 ± 0.4	58.3 ± 2.3	46.5 ± 0.1	43.1 ± 1.8	11.5 ± 0.2
	Day-14	44.6 ± 0	50.6 ± 1.1	50.8 ± 0.9	53.7 ± 0.2	58.8 ± 0.8	54.6 ± 1.5	39.6 ± 5.6	44.2 ± 3	6.3 ± 0.6
Pb	Day-7	0 ± 0	38.4 ± 0.5	5.7 ± 0.2	0 ± 0	36.7 ± 0.5	5.2 ± 0.1	0 ± 0	24.9 ± 1.1	1.4 ± 0.3
	Day-14	0 ± 0	45.7 ± 0.3	5.7 ± 0.2	0 ± 0	41 ± 0.5	5.5 ± 0.1	0 ± 0	28.8 ± 2.7	0.8 ± 0.2
Zn	Day-7	0.5 ± 0.1	25.8 ± 0.4	31.3 ± 0.2	0.6 ± 0	31.8 ± 0.1	33 ± 1.3	1.3 ± 0.1	43.3 ± 0.6	39.5 ± 0.5
	Day-14	0.4 ± 0.1	27 ± 0.3	36.6 ± 0.2	0.6 ± 0	32 ± 0.4	32.7 ± 0.9	0.9 ± 0	40.8 ± 1.2	35.5 ± 0.6
Co	Day-7	19.2 ± 1.1	41.2 ± 0.8	43.4 ± 0.3	19.8 ± 0.5	49.1 ± 0.3	49.2 ± 2.2	27.6 ± 0.3	32 ± 0.8	30.6 ± 1.2
	Day-14	16.5 ± 0.8	40.5 ± 0.8	41.9 ± 0.2	16.6 ± 0.4	46.4 ± 0.2	46.5 ± 1.3	23.2 ± 2.6	30.1 ± 1.2	29.3 ± 0
As	Day-7	0.1 ± 0	0.7 ± 0	0.4 ± 0.1	0.1 ± 0	0.2 ± 0	0 ± 0	2.4 ± 0.2	3.2 ± 0.1	0 ± 0
	Day-14	0.1 ± 0	0.4 ± 0	0.5 ± 0	0.2 ± 0	0.1 ± 0	0 ± 0	3.1 ± 0.4	0.9 ± 0.1	0 ± 0
Cd	Day-7	8.6 ± 0.3	31.6 ± 0	37.4 ± 0.8	11 ± 0.8	36.8 ± 0.5	38.1 ± 0.5	18.2 ± 0.3	46.8 ± 0.5	46.7 ± 1.6
	Day-14	7.5 ± 0.7	30.3 ± 0.2	39.9 ± 0.8	10.6 ± 0.7	34.8 ± 0.5	36.9 ± 0.9	11.3 ± 3.8	45.1 ± 1.3	41.8 ± 0.3
K	Day-7	0 ± 0	39.2 ± 0.7	4.9 ± 0	0 ± 0	0 ± 0	3.6 ± 0	0 ± 0	0 ± 0	2.8 ± 0
	Day-14	0 ± 0	37.6 ± 2.9	2.7 ± 0	0 ± 0	0 ± 0	3.5 ± 0.1	0 ± 0	0 ± 0	2.5 ± 0
Sb	Day-7	1.6 ± 0.1	0.5 ± 0	0.1 ± 0	0.8 ± 0	0.3 ± 0	0.1 ± 0	0.6 ± 0	0.7 ± 0.1	0.1 ± 0
	Day-14	3.1 ± 0.1	0.5 ± 0	0.1 ± 0	1.4 ± 0.1	0.3 ± 0	0.1 ± 0	0.7 ± 0	0.9 ± 0	0.1 ± 0
Ag	Day-7	36 ± 2.6	15.3 ± 1.1	0 ± 0	34.9 ± 2.5	27.1 ± 0.1	0 ± 0	12.2 ± 0.7	15.8 ± 1.4	0 ± 0
	Day-14	25.3 ± 0.3	10.6 ± 1.5	0 ± 0	29.6 ± 0.8	24.3 ± 0.5	0 ± 0	17.8 ± 0	15.4 ± 0.5	0 ± 0
Mn	Day-7	12.9 ± 0.4	30.1 ± 0.4	32.9 ± 0.4	21.1 ± 1	43.3 ± 0.4	44.7 ± 2	11.9 ± 0.5	43.5 ± 1	44.2 ± 1.2
	Day-14	11.2 ± 0.8	30.2 ± 0.6	30.9 ± 0.4	18.8 ± 1.2	41.3 ± 0.2	41.6 ± 1	9 ± 3.2	40.7 ± 1.8	42.1 ± 0.2
In	Day-7	0 ± 0	22.4 ± 1.8	0.9 ± 0	0 ± 0	27.4 ± 0.5	0.5 ± 0	0 ± 0	1.7 ± 0.4	0 ± 0
	Day-14	0 ± 0	22.6 ± 0.3	0.6 ± 0	0 ± 0	26.2 ± 0.7	0.5 ± 0	0 ± 0	1.3 ± 0.6	0 ± 0

**Table A10:** The distribution of elements in the minerals (groups) present in the Neves Corvo waste rock (NC_01).

	Elements (%)												
Mineral	Cu	Pb	Zn	Fe	S	Co	As	K	Mn	Sb	Al	Si	Ca
Silicates				26.3				100.0			99.9	100.0	1.4
Pyrite		10.1	3.6	58.2	94.0	16.0			15.5				
Arsenopyrite		0.1		0.3	0.2	3.2	67.9		0.2				
Chalcopyrite	91.2	0.2		1.6	2.5	0.3			0.6				
Covellite													
Galena		36.9			0.1	0.0			0.0				
Sphalerite		0.4	96.4	0.1	2.1	0.3			0.6				
Cobaltite					0.1	80.2	27.6						
Bornite	0.0			0.0	0.0								
Stannite	1.8			0.0	0.1								
Clausthalite		3.2											
Wittite		3.4			0.0								
Carbonates				8.1					2.7				46.5
Oxides		45.7		5.4					80.4		0.0		
Sulphates				0.0	0.9			0.0					38.1
Sulphosalts	7.0	0.1		0.0	0.1	0.0	4.5		0.0	100.0			
Phosphates											0.1		14.0

**Table A11:** Mineral compositions of the Neves Corvo waste rock (NC_01) before and after bioleaching obtained from the SEM/MLA-GXMAP.

Mineral (Groups) (wt%)	NC_01 Before Bioleaching	NC_01 After Bioleaching by:	
		*T. electrotropha*	*T. pacifica*
Silicates	78.9 ± 0.4	77.8 ± 0.5	80 ± 0.9
Pyrite	15.5 ± 0.3	15.8 ± 0.4	14.3 ± 0.3
Other sulphides	1.4 ± 0.1	1.6 ± 0.5	1.3 ± 0
Carbonates	2.6 ± 0.1	2.7 ± 0.4	2.3 ± 0.5
Oxides	1.3 ± 0.2	1.9 ± 0	1.7 ± 0.1
Sulphates	0.4 ± 0.1	0.1 ± 0	0.1 ± 0
Sulphosalts	0 ± 0	0 ± 0	0 ± 0
Phosphates	0.1 ± 0	0.1 ± 0	0.1 ± 0
Sum other minerals	0 ± 0	0 ± 0	0 ± 0

**Table A12:** Results from the XRD analysis of the Neves Corvo waste rock (NC_01) before and after microwave roasting at 400, 500 and 600 °C.

	Sum of % (m/m)								
Samples	Chlorite	Muscovite	Chamosite	Gypsum	Hematite	Pyrite	Quartz	Rutile	Siderite
NC_01	21 ± 0.5	17.9 ± 1				12.3 ± 0	45.6 ± 0.1	0.9 ± 0.1	2.7 ± 0
400 °C	12.5 ± 0.9	20.9 ± 0.4	2.6 ± 0.2	3 ± 0.1	2.8 ± 0	4.3 ± 0.1	52 ± 0.1	2 ± 0	
500 °C	5.7 ± 0.3	19.3 ± 0.2	3.7 ± 0.3	3.6 ± 0.1	4.5 ± 0.1	0.2 ± 0	60.3 ± 0.7	2.8 ± 0.1	
600 °C	4.1 ± 0.1	14.7 ± 0	2 ± 0.1	2.7 ± 0.4	10.7 ± 0.3	0.1 ± 0	63.1 ± 0.2	2.7 ± 0.1	

**Table A13:** Sulphur and hematite content of the Neves Corvo waste rock (NC_01) before and after microwave roasting obtained by X-ray fluorescence (XRF). Values are the average of duplicate samples ± standard deviations.

(%)	NC_01 Before Microwave-Roasting	NC_01 After Microwave-Roasting at 400 °C	NC_01 After Microwave-Roasting at 500 °C	NC_01 After Microwave-Roasting at 600 °C
S	4.8 ± 0.2	2.4 ± 0	2.2 ± 0	2.0 ± 0
