# Supplementary material for: Interesting Halophilic Sulphur-Oxidising Bacteria with Bioleaching Potential: Implications for Pollutant Mobilisation from Mine Waste
Source: Microorganisms. 2023 Jan 15;11(1):222. doi: 10.3390/microorganisms11010222 (PMC9866277; doi:10.3390/microorganisms11010222)
Supplement: Supplementary file 1 [file microorganisms-11-00222-s001.zip › microorganisms-2072315-supplementary.pdf]

Supplementary Material

Table S1. Concentrations of metal(loid)s extracted from the Neves Corvo waste rock by *T. cyclica* via one-step bioleaching (1SB), two-step bioleaching (2SB), spent medium bioleaching (SMB), and Abiotic control (AC). Values are the average of duplicate flasks ± standard deviations.

		Concentration (μg/L)			
**Metal(loid)s**	**Days**	**1SB**	**2SB**	**SMB**	**AC**
**Cu**	Day-7	11537.4 ± 70.7	1227.4 ± 28.3	1492.4 ± 35.4	13.9 ± 6.9
	Day-14	8487.4 ± 84.9	2887.4 ± 99	1472.4 ± 35.4	8 ± 2.1
	Day-21	7142.4 ± 35.4	3522.4 ± 77.8	1112.4 ± 7.1	18.2 ± 2.5
	Day-28	5517.4 ± 70.7	3707.4 ± 28.3	903.4 ± 90.5	16.6 ± 6.3
**Pb**	Day-7	1657.9 ± 254.6	2167.9 ± 14.1	2057.9 ± 198	5.4 ± 0.4
	Day-14	846.4 ± 36.1	714.9 ± 39.6	609.4 ± 62.9	5.4 ± 2
	Day-21	541.4 ± 34.6	293.4 ± 10.6	289.4 ± 7.8	6 ± 1
	Day-28	253.4 ± 7.8	149.9 ± 2.8	132.3 ± 63.1	4.7 ± 2.8
**Zn**	Day-7	2273.1 ± 834.4	149.6 ± 6.4	254.1 ± 17	440 ± 39.6
	Day-14	2033.1 ± 56.6	543.6 ± 46	249.1 ± 21.2	778 ± 384.7
	Day-21	2073.1 ± 28.3	724.1 ± 36.8	312.6 ± 12	748 ± 193.7
	Day-28	1468.1 ± 247.5	868.1 ± 53.7	173.1 ± 11.3	1379.5 ± 806.8
**Co**	Day-7	1.5 ± 8.5	101.5 ± 5.7	119 ± 2.1	71.5 ± 7.7
	Day-14	30 ± 0.7	143 ± 7.8	218 ± 13.4	101.8 ± 35.7
	Day-21	58.5 ± 4.2	201.5 ± 12.7	292.5 ± 15.6	100.3 ± 23.7
	Day-28	89.5 ± 4.2	261 ± 9.2	255 ± 6.4	152.5 ± 72.8
**As**	Day-7	15700 ± 989.9	6370 ± 155.6	10850 ± 70.7	<10
	Day-14	17100 ± 282.8	12700 ± 141.4	13850 ± 495	28 ± 12.7
	Day-21	17700 ± 707.1	15350 ± 70.7	13850 ± 495	<100
	Day-28	16950 ± 1060.7	16250 ± 70.7	12800 ± 0	<100
**Cd**	Day-7	18.3 ± 1.5	2.8 ± 0.3	3.8 ± 0.2	6.1 ± 0.9
	Day-14	14 ± 0.8	4.3 ± 0.3	3 ± 0.6	8.7 ± 2.8
	Day-21	14.1 ± 0.7	5 ± 0.2	2.9 ± 0.3	9.2 ± 2.2
	Day-28	10.4 ± 1.4	4.9 ± 0.4	2.1 ± 0.5	12.3 ± 4.1
**K**	Day-7	225500 ± 7778.2	203000 ± 0	44000 ± 8485	13000 ± 848.5
	Day-14	90500 ± 6364	70500 ± 6364	0 ± 0	11550 ± 70.7
	Day-21	120500 ± 6364	80500 ± 9192	358000 ± 33941	14500 ± 2262.7
	Day-28	68000 ± 5656.9	43500 ± 6364	126000 ± 4242.6	12850 ± 1060.7
**Sb**	Day-7	597 ± 12	145.5 ± 8.5	271.5 ± 9.9	8.9 ± 0.9
	Day-14	711.5 ± 22.6	325.5 ± 14.1	374 ± 3.5	6.9 ± 1
	Day-21	827.5 ± 15.6	461 ± 23.3	501 ± 13.4	6.7 ± 0.6
	Day-28	924 ± 12	579 ± 27.6	478 ± 0.7	5 ± 1.2
**Ag**	Day-7	126 ± 1.4	<1	3.6 ± 1.4	<1
	Day-14	129 ± 1.4	13 ± 0.9	<1	<1
	Day-21	96.5 ± 3.3	50.9 ± 2.3	2.1 ± 0.1	10.8 ± 1.3
	Day-28	67.5 ± 6.2	50.6 ± 2.1	<1	6.9 ± 2.7
**Mn**	Day-7	29.6 ± 4.7	3.3 ± 0.4	0 ± 0	249 ± 21.2
	Day-14	0 ± 0	0 ± 0	0 ± 0	3090 ± 353.6
	Day-21	0 ± 0	0 ± 0	0 ± 0	3310 ± 438.4
	Day-28	0 ± 0	0 ± 0	0 ± 0	3665 ± 700

Table S2. Concentrations of metal(loid)s extracted from the Neves Corvo waste rock by *T. thiocyanaticus* via one-step bioleaching (1SB), two-step bioleaching (2SB), spent medium bioleaching (SMB), and Abiotic control (AC). Values are the average of duplicate flasks ± standard deviations.

		Concentration (µg/L)			
**Metal(loid)s**	**Days**	**1SB**	**2SB**	**SMB**	**AC**
**Cu**	Day-7	6674.7 ± 70.7	960.7 ± 19.8	922.2 ± 60.1	11.2 ± 9.3
	Day-14	2249.7 ± 7.1	1319.7 ± 91.9	868.7 ± 101.8	31.4 ± 19
	Day-21	1734.7 ± 56.6	1814.7 ± 127.3	733.7 ± 99	19.2 ± 1.2
	Day-28	1524.7 ± 14.1	1964.7 ± 410.1	790.7 ± 89.1	6.1 ± 1.1
**Pb**	Day-7	114.9 ± 11.3	122.4 ± 2.1	175.4 ± 17.7	4 ± 1.3
	Day-14	58.1 ± 2.4	54.5 ± 6.6	36.5 ± 13.9	4.9 ± 1.4
	Day-21	35.2 ± 7.1	33.4 ± 0.7	15.1 ± 4.5	3.2 ± 0.4
	Day-28	39.6 ± 6.7	43 ± 3.7	11.5 ± 3.3	3.1 ± 0.3
**Zn**	Day-7	213.4 ± 18.4	55.5 ± 28.1	97.9 ± 4.9	289 ± 28.3
	Day-14	84.4 ± 11.3	130.4 ± 5.7	42.7 ± 3.8	338 ± 63.6
	Day-21	109.9 ± 9.2	19.4 ± 10.2	27 ± 34.4	308.5 ± 53
	Day-28	89.9 ± 3.5	5.2 ± 6.8	43.8 ± 20.6	369.5 ± 171.8
**Co**	Day-7	56.9 ± 1.4	53.9 ± 1.4	32.8 ± 3.4	51.5 ± 3.5
	Day-14	47.9 ± 4.2	13.3 ± 7.4	0 ± 0	51.5 ± 16.3
	Day-21	45.4 ± 0.7	0 ± 0	24.4 ± 10.5	39.3 ± 10.2
	Day-28	41.5 ± 10.5	0 ± 0	25.1 ± 7	51.6 ± 23.5
**As**	Day-7	280.5 ± 51.6	418 ± 15.6	432.5 ± 12	<10
	Day-14	1035 ± 35.4	554 ± 8.5	604 ± 12.7	<100
	Day-21	1009.5 ± 43.1	611.5 ± 13.4	596.5 ± 14.8	<100
	Day-28	1032.5 ± 95.5	551.5 ± 123.7	646.5 ± 0.7	<100
**Cd**	Day-7	14.7 ± 1.8	12.2 ± 0.1	14.5 ± 2.1	4.5 ± 0.4
	Day-14	8.3 ± 0.4	6 ± 0.4	14.3 ± 4	4.6 ± 1.6
	Day-21	10.1 ± 0.1	5.6 ± 0.7	11.6 ± 3.3	4.1 ± 0.9
	Day-28	8 ± 1	5.7 ± 1.2	10.7 ± 3.1	4.6 ± 1.4
**K**	Day-7	37500 ± 2121.3	32000 ± 5656.9	0 ± 0	11200 ± 565.7
	Day-14	52000 ± 2828.4	39000 ± 5656.9	366500 ± 50204.6	11900 ± 141.4
	Day-21	43000 ± 11313.7	45500 ± 707.1	85500 ± 28991.4	12450 ± 212.1
	Day-28	0 ± 0	0 ± 0	149000 ± 33941.1	9590 ± 325.3
**Sb**	Day-7	597 ± 12	145.5 ± 8.5	271.5 ± 9.9	8.9 ± 0.9
	Day-14	711.5 ± 22.6	325.5 ± 14.1	374 ± 3.5	6.9 ± 1
	Day-21	827.5 ± 15.6	461 ± 23.3	115.5 ± 11.3	6.7 ± 0.6
	Day-28	924 ± 12	579 ± 27.6	114 ± 10.6	5 ± 1.2
**Ag**	Day-7	40.5 ± 4.4	<1	0.3 ± 0	<1
	Day-14	90.6 ± 1.8	57.3 ± 4.8	4.2 ± 2.3	9.8 ± 0.9
	Day-21	42.8 ± 4.6	32 ± 1.3	4.5 ± 2.9	6.6 ± 3.3
	Day-28	64.6 ± 1.1	38.7 ± 8.2	4.5 ± 2.9	3 ± 1
**Mn**	Day-7	0 ± 0	4.7 ± 2.4	95.5 ± 14.1	2330 ± 127.3
	Day-14	0 ± 0	0 ± 0	143 ± 26.2	2390 ± 268.7
	Day-21	0 ± 0	0 ± 0	98.5 ± 21.2	2230 ± 367.7
	Day-28	0 ± 0	0 ± 0	119.5 ± 19.8	2425 ± 558.6

Table S3. Concentrations of metal(loid)s extracted from the Neves Corvo waste rock by *T. electrotropha* via one-step bioleaching (1SB), two-step bioleaching (2SB), spent medium bioleaching (SMB), and Abiotic control (AC). Values are the average of duplicate flasks ± standard deviations.

		**Concentration (μg/L)**			
**Metal(loid)s**	**Days**	**1SB**	**2SB**	**SMB**	**AC**
**Cu**	Day-7	2000 ± 226.3	1615 ± 233.3	1660 ± 254.6	3.8 ± 0.6
	Day-14	4525 ± 233.3	3600 ± 254.6	2050 ± 169.7	9.4 ± 2.3
	Day-21	6200 ± 339.4	4125 ± 346.5	4085 ± 700	12.6 ± 0.8
	Day-28	8010 ± 664.7	6425 ± 1449.6	6685 ± 912.2	22.6 ± 0.7
**Pb**	Day-7	190.8 ± 23.3	1357.8 ± 522.6	6.8 ± 2.1	2.9 ± 0.1
	Day-14	3102.3 ± 77.8	5667.3 ± 325.3	6.5 ± 0.3	2.6 ± 0.4
	Day-21	3917.3 ± 28.3	4077.3 ± 42.4	4137.3 ± 1074.8	2.4 ± 1.1
	Day-28	4067.3 ± 155.6	3957.3 ± 14.1	5122.3 ± 275.8	2.4 ± 0.4
**Zn**	Day-7	8893.7 ± 516.2	13058.7 ± 636.4	220.7 ± 50.9	434 ± 113.1
	Day-14	14958.7 ± 353.6	15608.7 ± 141.4	25 ± 16.5	597.5 ± 200.1
	Day-21	18408.7 ± 141.4	16508.7 ± 424.3	15758.7 ± 1626.3	600.5 ± 277.9
	Day-28	21808.7 ± 707.1	18408.7 ± 1838.5	22758.7 ± 3747.7	1300 ± 113.1
**Co**	Day-7	954.5 ± 4.9	963 ± 9.9	408 ± 26.9	65.3 ± 10.2
	Day-14	1025 ± 7.1	1065 ± 21.2	318.5 ± 40.3	76 ± 14.7
	Day-21	1060 ± 28.3	1019.5 ± 57.3	887.5 ± 102.5	61.6 ± 22.1
	Day-28	1055 ± 35.4	995.5 ± 62.9	1032.5 ± 81.3	82.3 ± 13.4
**As**	Day-7	187 ± 24	216.5 ± 21.9	116.5 ± 10.6	<10
	Day-14	359.5 ± 10.6	277 ± 1.4	157 ± 29.7	<10
	Day-21	698 ± 56.6	1250 ± 212.1	250.5 ± 40.3	<10
	Day-28	1265 ± 77.8	1840 ± 282.8	814.5 ± 516.9	<10
**Cd**	Day-7	59.7 ± 1.7	59.6 ± 1.6	21.4 ± 3.6	4.7 ± 1.1
	Day-14	63.5 ± 2.4	59.3 ± 1.9	9.6 ± 1.2	5.7 ± 1.6
	Day-21	63.7 ± 3.6	58.1 ± 3	56.9 ± 4	5.5 ± 2
	Day-28	72.1 ± 5.2	61.7 ± 8.1	76.3 ± 11.1	10 ± 1.6
**K**	Day-7	0 ± 0	0 ± 0	43500 ± 5656.9	14800 ± 282.8
	Day-14	0 ± 0	0 ± 0	0 ± 0	14200 ± 282.8
	Day-21	66500 ± 2828.4	55500 ± 2828.4	0 ± 0	15250 ± 2192
	Day-28	0 ± 0	0 ± 0	152000 ± 72832	15300 ± 282.8
**Sb**	Day-7	28.4 ± 1.2	20 ± 1.3	28.3 ± 0.1	16.6 ± 0.2
	Day-14	38.5 ± 1.4	21.7 ± 1	52.8 ± 1.5	16.3 ± 0.4
	Day-21	38.9 ± 0.1	31.9 ± 4	34.9 ± 8.8	21.8 ± 0.4
	Day-28	51.2 ± 6.1	42.2 ± 12.6	37.9 ± 6.7	50.8 ± 20.9
**Ag**	Day-7	<1	2.1 ± 1.4	<1	<1
	Day-14	9.4 ± 0.5	14 ± 1.5	2.4 ± 0.3	<1
	Day-21	8.8 ± 0.8	15.2 ± 2.8	13.5 ± 6.1	<1
	Day-28	11.4 ± 1.1	10.3 ± 0.9	14.7 ± 3.5	<1
**Mn**	Day-7	4782.3 ± 49.5	4562.3 ± 7.1	2772.3 ± 106.1	2000 ± 113.1
	Day-14	4867.3 ± 14.1	5162.3 ± 63.6	1242.3 ± 190.9	2240 ± 198
	Day-21	4937.3 ± 226.3	4957.3 ± 226.3	4272.3 ± 714.2	2200 ± 537.4
	Day-28	4652.3 ± 63.6	4582.3 ± 162.6	5047.3 ± 254.6	2545 ± 289.9

Table S4. Concentrations of metal(loid)s extracted from the Neves Corvo waste rock by *T. pacifica* via one-step bioleaching (1SB), two-step bioleaching (2SB), spent medium bioleaching (SMB), and Abiotic control (AC). Values are the average of duplicate flasks ± standard deviations.

		**Concentration (μg/L)**			
**Metal(loid)s**	**Days**	**1SB**	**2SB**	**SMB**	**AC**
**Cu**	Day-7	2154.1 ± 91.9	1229.1 ± 141.4	1874.1 ± 35.4	3.8 ± 0.6
	Day-14	4559.1 ± 707.1	1719.1 ± 169.7	2229.1 ± 70.7	9.4 ± 2.3
	Day-21	6014.1 ± 756.6	3369.1 ± 325.3	3984.1 ± 21.2	12.6 ± 0.8
	Day-28	7414.1 ± 431.3	4619.1 ± 1032.4	6694.1 ± 176.8	22.6 ± 0.7
**Pb**	Day-7	123.5 ± 42.1	1862.3 ± 21.2	120.8 ± 2.1	2.9 ± 0.1
	Day-14	3657.3 ± 707.1	140.3 ± 17	112.3 ± 8.5	2.6 ± 0.4
	Day-21	4072.3 ± 388.9	3602.3 ± 459.6	5282.3 ± 91.9	2.4 ± 1.1
	Day-28	4072.3 ± 318.2	4157.3 ± 707.1	5387.3 ± 169.7	2.4 ± 0.4
**Zn**	Day-7	3943.7 ± 1492	12008.7 ± 141.4	3828.7 ± 240.4	434 ± 113.1
	Day-14	16058.7 ± 1767.8	1228.7 ± 424.3	1218.7 ± 367.7	597.5 ± 200.1
	Day-21	19408.7 ± 3394.1	14408.7 ± 0	16458.7 ± 70.7	600.5 ± 277.9
	Day-28	21558.7 ± 2333.5	16758.7 ± 1343.5	22458.7 ± 1767.8	1300 ± 113.1
**Co**	Day-7	796.5 ± 60.1	925 ± 7.1	884 ± 11.3	65.3 ± 10.2
	Day-14	1095 ± 7.1	578.5 ± 27.6	563 ± 49.5	76 ± 14.7
	Day-21	1130 ± 42.4	1004 ± 22.6	1010 ± 14.1	61.6 ± 22.1
	Day-28	1100 ± 0	1020 ± 14.1	1130 ± 42.4	82.3 ± 13.4
**As**	Day-7	315.5 ± 4.9	250.5 ± 3.5	133.5 ± 4.9	<10
	Day-14	405 ± 75	207 ± 2.8	141.5 ± 4.9	<10
	Day-21	610.5 ± 190.2	242 ± 19.8	277.5 ± 2.1	<10
	Day-28	1039 ± 383.3	668 ± 83.4	952.5 ± 194.5	<10
**Cd**	Day-7	55.6 ± 2.6	60.7 ± 0.5	48.1 ± 0.4	4.7 ± 1.1
	Day-14	68 ± 3.7	32.7 ± 3.9	31.3 ± 3.5	5.7 ± 1.6
	Day-21	67.6 ± 4.7	54.3 ± 0.2	57.4 ± 1.6	5.5 ± 2
	Day-28	75.6 ± 3.9	59 ± 2	74.4 ± 6.3	10 ± 1.6
**K**	Day-7	0 ± 0	0 ± 0	38000 ± 4949.7	14800 ± 282.8
	Day-14	0 ± 0	0 ± 0	0 ± 0	14200 ± 282.8
	Day-21	56500 ± 4242.6	49500 ± 8485.3	0 ± 0	15250 ± 2192
	Day-28	0 ± 0	0 ± 0	67000 ± 14849.2	15300 ± 282.8
**Sb**	Day-7	38.5 ± 4.2	21.3 ± 0.4	26.7 ± 0.7	16.6 ± 0.2
	Day-14	34.8 ± 1.5	29.9 ± 0.2	47.1 ± 1.9	16.3 ± 0.4
	Day-21	38.9 ± 2	22.5 ± 1.5	27.3 ± 0.8	21.8 ± 0.4
	Day-28	51.9 ± 1	26.9 ± 4	38.3 ± 6.9	50.8 ± 20.9
**Ag**	Day-7	<1	<1	7.8 ± 1.6	<1
	Day-14	6 ± 0.1	<1	<1	<1
	Day-21	10.3 ± 0.8	8.9 ± 0.1	14.3 ± 4.5	<1
	Day-28	12.5 ± 1.9	8.1 ± 0.6	10.9 ± 0.4	<1
**Mn**	Day-7	3557.3 ± 509.1	4277.3 ± 113.1	4152.3 ± 21.2	2000 ± 113.1
	Day-14	4242.3 ± 318.2	2557.3 ± 183.8	2622.3 ± 304.1	2240 ± 198
	Day-21	4397.3 ± 141.4	4182.3 ± 134.4	4427.3 ± 169.7	2200 ± 537.4
	Day-28	4097.3 ± 226.3	4242.3 ± 162.6	4827.3 ± 127.3	2545 ± 289.9

Table S5. Concentrations of metal(loid)s extracted from the Neves Corvo waste rock by the *Thioclava* consortium via one-step bioleaching (1SB), two-step bioleaching (2SB), and spent medium bioleaching (SMB). Values are the average of duplicate flasks ± standard deviations.

		**Concentration (µg/L)**		
**Metal(loid)s**	**Days**	**1SB**	**2SB**	**SMB**
**Cu**	Day-7	2904.1 ± 63.6	1684.1 ± 459.6	1549.1 ± 678.8
	Day-14	4469.1 ± 42.4	1944.1 ± 134.4	2259.1 ± 183.8
	Day-21	6409.1 ± 42.4	3334.1 ± 120.2	4424.1 ± 728.3
	Day-28	8444.1 ± 685.9	4869.1 ± 466.7	8309.1 ± 1400.1
**Pb**	Day-7	390.3 ± 0	302.3 ± 0	408.3 ± 0
	Day-14	920.8 ± 475.9	4.3 ± 2.1	1445.8 ± 2038.6
	Day-21	4282.3 ± 63.6	4037.3 ± 198	5332.3 ± 629.3
	Day-28	4427.3 ± 339.4	5527.3 ± 749.5	7682.3 ± 323.2
**Zn**	Day-7	12408.7 ± 2121.3	12858.7 ± 3606.2	13908.7 ± 1838.5
	Day-14	12758.7 ± 1343.5	13041.7 ± 0	14163.2 ± 77.1
	Day-21	17958.7 ± 1626.3	15558.7 ± 495	17708.7 ± 1131.4
	Day-28	22958.7 ± 2616.3	18208.7 ± 141.4	24358.7 ± 3747.7
**Co**	Day-7	1045 ± 21.2	1002.5 ± 81.3	1024 ± 79.2
	Day-14	933.5 ± 37.5	288 ± 53.7	1070 ± 0
	Day-21	1022.5 ± 38.9	917.5 ± 21.9	1110 ± 70.7
	Day-28	1100 ± 56.6	992.5 ± 38.9	1255 ± 148.5
**As**	Day-7	774.5 ± 85.6	186.5 ± 24.7	306 ± 182.4
	Day-14	486 ± 1.4	144.5 ± 20.5	227 ± 134.4
	Day-21	651 ± 69.3	218 ± 72.1	1494.5 ± 176.1
	Day-28	1044 ± 79.2	763 ± 181	916 ± 345.1
**Cd**	Day-7	54.6 ± 0.7	53.8 ± 1.9	48.7 ± 1.3
	Day-14	56.2 ± 1.3	11.9 ± 2.3	58.4 ± 0
	Day-21	65 ± 4.2	56.5 ± 0.1	62.8 ± 2.9
	Day-28	77.4 ± 10.3	63.1 ± 1.8	90.7 ± 13.2
**K**	Day-7	26000 ± 7778.2	24000 ± 707.1	0 ± 0
	Day-14	0 ± 0	0 ± 0	55000 ± 7778.2
	Day-21	0 ± 0	0 ± 0	122000 ± 89802.6
	Day-28	45000 ± 3535.5	44500 ± 7071.1	142000 ± 9192.4
**Sb**	Day-7	31.6 ± 2.5	23.2 ± 8.1	40.3 ± 2.3
	Day-14	48.4 ± 5.7	46 ± 8.4	60.1 ± 11.7
	Day-21	47.4 ± 4.4	32 ± 2.5	45.1 ± 1.1
	Day-28	59.4 ± 9.7	30.4 ± 2.4	75.5 ± 1.2
**Ag**	Day-7	<1	3.1 ± 1.1	2.8 ± 0.4
	Day-14	8.4 ± 1.3	<1	5.1 ± 0
	Day-21	11.2 ± 1.5	15.6 ± 2.9	13.6 ± 5.2
	Day-28	10.7 ± 2	17.3 ± 0.1	8.8 ± 1.4
**Mn**	Day-7	5342.3 ± 77.8	4742.3 ± 275.8	4937.3 ± 14.1
	Day-14	4677.3 ± 339.4	1562.3 ± 190.9	5117.3 ± 0
	Day-21	4917.3 ± 381.8	4437.3 ± 56.6	5272.3 ± 304.1
	Day-28	5092.3 ± 431.3	5017.3 ± 14.1	5332.3 ± 205.1

Table S6. Concentrations of metal(loid)s extracted from the Neves Corvo waste rock (NC_01) via one-step bioleaching and two-step bioleaching by *T. electrotropha* (E-1SB & E-2SB), as well as one-step bioleaching and two-step bioleaching by *T. pacifica* (P-1SB & P-2SB). Results of weekly serial addition of NC_01 and partial nutrient replacement. Values are the average of duplicate flasks ± standard deviations.

		Concentration (μg/L)			
	**Days**	**E-1SB**	**E-2SB**	**P-1SB**	**P-2SB**
**Cu**	Day-7	931.1 ± 2.8	691.6 ± 235.5	892.6 ± 14.8	434.6 ± 64.3
	Day-14	1319.1 ± 84.9	1299.1 ± 84.9	2144.1 ± 855.6	1244.1 ± 120.2
	Day-21	4479.1 ± 466.7	2179.1 ± 311.1	2529.1 ± 0	2924.1 ± 1180.9
	Day-28	5554.1 ± 332.3	3554.1 ± 49.5	3559.1 ± 0	2849.1 ± 0
	Day-35	4884.1 ± 912.2	4124.1 ± 247.5	4509.1 ± 0	7434.1 ± 544.5
	Day-42	4379.1 ± 466.7	3719.1 ± 664.7	5329.1 ± 1032.4	5794.1 ± 275.8
**Pb**	Day-7	684.3 ± 166.9	1712.3 ± 64.35	191.3 ± 0	196.3 ± 0
	Day-14	128.3 ± 181	2.1 ± 0.6	830.3 ± 844.3	130.3 ± 18.4
	Day-21	3962.3 ± 898	3127.3 ± 70.7	488.3 ± 425.7	451.3 ± 76.4
	Day-28	2577.3 ± 183.8	2252.3 ± 261.6	2022.3 ± 601	1167.3 ± 1131.4
	Day-35	1472.3 ± 374.8	1642.3 ± 148.5	1492.3 ± 318.2	1637.3 ± 495
	Day-42	1087.3 ± 84.9	1322.3 ± 332.3	1012.3 ± 233.3	1138.8 ± 323.1
**Zn**	Day-7	3598.7 ± 0	3638.7 ± 99	2559.7 ± 114.6	2623.7 ± 799
	Day-14	4298.7 ± 0	0 ± 0	454.7 ± 0	219.7 ± 22.6
	Day-21	17108.7 ± 0	14408.7 ± 141.4	16653.2 ± 219.9	12208.7 ± 2404
	Day-28	16758.7 ± 70.7	12958.7 ± 212.1	18058.7 ± 4313.4	18508.7 ± 933
	Day-35	13359 ± 2758	11458.7 ± 777.8	15208.7 ± 989.9	18258.7 ± 1626
	Day-42	11358.7 ± 636	10173.7 ± 1181	12408.7 ± 282.8	12408.7 ± 707.1
**Co**	Day-7	263.5 ± 0.7	259.5 ± 6.4	255 ± 18.4	250 ± 29.7
	Day-14	332 ± 0	234.5 ± 26.2	218.5 ± 6.4	196.5 ± 55.9
	Day-21	881.5 ± 7.8	796.5 ± 17.7	688.5 ± 94	657 ± 1.4
	Day-28	690 ± 8.5	642.5 ± 0.7	710 ± 2.8	541 ± 148.5
	Day-35	465.5 ± 65.8	455 ± 14.1	557 ± 25.5	516 ± 32.5
	Day-42	319 ± 21.2	302.5 ± 38.9	394.5 ± 4.9	362 ± 12.7
**As**	Day-7	379 ± 9.9	257.5 ± 38.9	338.5 ± 19.1	156 ± 18.4
	Day-14	377.5 ± 37.5	344 ± 43.8	236 ± 93.3	265 ± 14.1
	Day-21	1516 ± 76.4	419.5 ± 24.7	408 ± 50.9	341.5 ± 98.3
	Day-28	2545 ± 162.6	1990 ± 127.3	663.5 ± 320.3	411.5 ± 78.5
	Day-35	2730 ± 127.3	2595 ± 49.5	2498.5 ± 535.3	543 ± 99
	Day-42	2435 ± 558.6	2160 ± 282.8	2340 ± 56.6	827 ± 43.8
**Cd**	Day-7	11.1 ± 0.2	10.8 ± 0.4	9.8 ± 1.3	9.4 ± 0.8
	Day-14	7.6 ± 0	5.4 ± 0.5	12.5 ± 0	9.6 ± 0
	Day-21	55.5 ± 5.3	52.9 ± 0.1	69.3 ± 0	61.9 ± 8.8
	Day-28	50.1 ± 1.4	41.5 ± 1.8	54.4 ± 11.1	63.5 ± 1.7
	Day-35	38.7 ± 6.9	33.8 ± 2.7	42.2 ± 2.1	46.9 ± 0.9
	Day-42	36 ± 0.4	32.4 ± 3.7	37.6 ± 1.6	34.8 ± 2.5
**K**	Day-7	25500 ± 7071	27500 ± 0	22500 ± 4242.6	16500 ± 1414.2
	Day-14	0 ± 0	0 ± 0	0 ± 0	0 ± 0
	Day-21	0 ± 0	0 ± 0	0 ± 0	0 ± 0
	Day-28	51500 ± 0	52000 ± 6364	48000 ± 707.1	44000 ± 707.1
	Day-35	41500 ± 0	47500 ± 1414.2	44500 ± 5656.9	34000 ± 2121.3
	Day-42	123500 ± 4243	116000 ± 7778	116000 ± 3535.5	100500 ± 15556
**Sb**	Day-7	11.4 ± 0.5	16.3 ± 2.2	12.4 ± 0.1	7.9 ± 0.2
	Day-14	41.8 ± 3.9	49.6 ± 5.8	30.4 ± 12.2	38.8 ± 6

	Day-21	48.8 ± 0	20.8 ± 2	49.4 ± 0	66.6 ± 0
	Day-28	36.9 ± 9.3	20.1 ± 0.1	22.9 ± 0	104 ± 0
	Day-35	36.5 ± 3.8	20.2 ± 1.1	24.1 ± 0	41.2 ± 0
	Day-42	39.1 ± 14.6	25.3 ± 1.8	29.6 ± 0	31.7 ± 0
**Ag**	Day-7	3.9 ± 0.4	2.3 ± 0.6	1.9 ± 0.3	<1
	Day-14	6 ± 0	6.7 ± 0	1.9 ± 0	<1
	Day-21	8.6 ± 1.8	10.3 ± 1.4	32.1 ± 1.6	33.3 ± 0
	Day-28	8.8 ± 1	9.2 ± 0.4	6.2 ± 0	11.6 ± 0
	Day-35	11.5 ± 2	10.8 ± 1.1	6.6 ± 0	33.1 ± 3.4
	Day-42	10.7 ± 2.3	15.8 ± 6	7.3 ± 0	23.9 ± 5.6
**Mn**	Day-7	1307.3 ± 14.1	1332.3 ± 63.6	981.8 ± 6.4	912.8 ± 190.2
	Day-14	1755.8 ± 115.3	537.8 ± 146.4	2727.3 ± 0	312.3 ± 103.2
	Day-21	4297.3 ± 113.1	3947.3 ± 282.8	2937.3 ± 0	2802.3 ± 21.2
	Day-28	3127.3 ± 155.6	3002.3 ± 106.1	2837.3 ± 594	1922.3 ± 403.1
	Day-35	1977.3 ± 282.8	1952.3 ± 77.8	2062.3 ± 388.9	1852.3 ± 219.2
	Day-42	1262.3 ± 91.9	1187.3 ± 99	1397.3 ± 56.6	1377.3 ± 226.3

Table S7. Concentrations of metal(loid)s extracted from the microwave-roasted (at 400, 500 and 600°C) Neves Corvo waste rock (NC_01) by *T. electrotropha* via one-step bioleaching (1SB), two-step bioleaching (2SB) and Abiotic control (AC). Values are the average of duplicate flasks ± standard deviations.

		Concentration (μg/L)								
	**Days**	**400°C 1SB**	**400°C 2SB**	**400°C AC**	**500°C 1SB**	**500°C 2SB**	**500°C AC**	**600°C 1SB**	**600°C 2SB**	**600°C AC**
**Cu**	Day-7	42539.1 ± 70.7	45350 ± 777.8	47800 ± 424.3	54439.1 ± 495	56439.1 ± 353.6	53600 ± 2121.3	42739.1 ± 70.7	39639.1 ± 1626.3	10600 ± 141.4
	Day-14	40989.1 ± 0	46589.1 ± 989.9	46700 ± 848.5	49389.1 ± 141.4	54089.1 ± 707.1	50250 ± 1343.5	36439.1 ± 5161.9	40639.1 ± 2757.7	5770 ± 509.1
**Pb**	Day-7	0 ± 0	15850 ± 212.1	2355 ± 77.8	0 ± 0	15147.3 ± 212.1	2150 ± 28.3	7.3 ± 1.1	10282.3 ± 445.5	568.5 ± 115.3
	Day-14	0.4 ± 0.1	18900 ± 141.4	2370 ± 70.7	0 ± 0	16947.3 ± 212.1	2290 ± 42.4	11.3 ± 5.4	11897.3 ± 1131.4	313 ± 70.7
**Zn**	Day-7	839.2 ± 89.8	45950 ± 777.8	55750 ± 353.6	1003.7 ± 21.2	56608.7 ± 141.4	58800 ± 2262.7	2313.7 ± 134.4	77058.7 ± 1060.7	70350 ± 919.2
	Day-14	733.2 ± 112.4	44800 ± 565.7	60750 ± 353.6	1058.7 ± 56.6	53558.7 ± 636.4	54750 ± 1484.9	1578.7 ± 0	71858.7 ± 2192	62500 ± 989.9
**Co**	Day-7	812.5 ± 46	1745 ± 35.4	1840 ± 14.1	837.5 ± 21.9	2080 ± 14.1	2085 ± 91.9	1170 ± 14.1	1355 ± 35.4	1295 ± 49.5
	Day-14	698.5 ± 33.2	1715 ± 35.4	1775 ± 7.1	704 ± 18.4	1965 ± 7.1	1970 ± 56.6	982.5 ± 109.6	1275 ± 49.5	1240 ± 0
**As**	Day-7	40.4 ± 5.6	266 ± 18.4	145.5 ± 31.8	52.3 ± 10	67.6 ± 6.9	14.1 ± 1.7	923.5 ± 89.8	1220 ± 28.3	16.7 ± 1.1
	Day-14	53.7 ± 6.3	156 ± 2.8	179.5 ± 7.8	70 ± 6.9	31.2 ± 0.6	18.1 ± 0.1	1180 ± 169.7	329 ± 24	18.1 ± 0.4
**Cd**	Day-7	38.9 ± 1.1	142 ± 0	168.5 ± 3.5	49.7 ± 3.7	165.5 ± 2.1	171.5 ± 2.1	81.9 ± 1.4	210.5 ± 2.1	210 ± 7.1
	Day-14	33.9 ± 3.3	136.5 ± 0.7	179.5 ± 3.5	47.7 ± 3.2	156.5 ± 2.1	166 ± 4.2	51.1 ± 17.3	203 ± 5.7	188 ± 1.4
**K**	Day-7	0 ± 0	283500 ± 4949.7	35700 ± 0	0 ± 0	0 ± 0	26200 ± 0	0 ± 0	0 ± 0	20100 ± 141.4
	Day-14	0 ± 0	272000 ± 21213.2	19700 ± 0	0 ± 0	0 ± 0	25200 ± 707.1	0 ± 0	0 ± 0	18150 ± 70.7
**Sb**	Day-7	38.1 ± 1.8	10.9 ± 0.4	2.3 ± 0.2	19.6 ± 1.1	7.1 ± 0	2 ± 0	15.3 ± 0.6	17.2 ± 1.3	3.5 ± 0
	Day-14	73.7 ± 2.1	11.5 ± 0.1	1.8 ± 0.2	33.5 ± 2	6.5 ± 0.1	1.4 ± 0.1	17.8 ± 1	20.7 ± 1.1	3.5 ± 0.2
**Ag**	Day-7	97.8 ± 0.6	39.8 ± 2.8	1.6 ± 0	91.6 ± 6.5	71.2 ± 0.3	<1	34.5 ± 1.9	44.5 ± 3.8	<1
	Day-14	65.8 ± 0.8	27.6 ± 3.8	<1	77.7 ± 2.1	63.7 ± 1.3	<1	50.1 ± 0	43.5 ± 1.5	<1
**Mn**	Day-7	4662.3 ± 162.6	10900 ± 141.4	11900 ± 141.4	7627.3 ± 367.7	15697.3 ± 141.4	16200 ± 707.1	4297.3 ± 169.7	15747.3 ± 353.6	16000 ± 424.3
	Day-14	4052.3 ± 304.1	10950 ± 212.1	11200 ± 141.4	6822.3 ± 445.5	14947.3 ± 70.7	15050 ± 353.6	3272.3 ± 1152.6	14747.3 ± 636.4	15250 ± 70.7
**In**	Day-7	<1	56.2 ± 4.5	2.3 ± 0.1	<1	67.7 ± 1.3	1.2 ± 0.1	<1	4.5 ± 1.1	<1
	Day-14	<1	56.7 ± 0.6	1.6 ± 0	<1	64.7 ± 1.8	1.2 ± 0.1	<1	3.4 ± 1.6	<1

Table S8. Concentrations and percentages of metal(loid)s extracted from the Neves Corvo waste rock (NC_01) by the second experiment with *T. electrotropha* via one-step bioleaching (1SB), two-step bioleaching (2SB), spent medium bioleaching (SMB), and Abiotic control (AC). Values are the average of duplicate flasks ± standard deviations.

		Concentration (µg/L)				% Recovery			
**Metal(loid)s**	**Days**	**1SB**	**2SB**	**SMB**	**AC**	**1SB**	**2SB**	**SMB**	**AC**
**Cu**	Day-7	975.6 ± 188.8	1034.1 ± 7.1	3.6 ± 0.1	3.8 ± 0.6	1.1 ± 0.2	1.1 ± 0	0 ± 0	0 ± 0
	Day-21	4744.1 ± 2213.2	1534.1 ± 601	1519.1 ± 56.6	9.4 ± 2.3	5.2 ± 2.4	1.7 ± 0.7	1.7 ± 0.1	0 ± 0
	Day-28	7779.1 ± 2828.4	3794.1 ± 855.6	1818.7 ± 84.4	12.6 ± 0.8	8.5 ± 3.1	4.1 ± 0.9	2 ± 0.1	0 ± 0
**Pb**	Day-7	1739.3 ± 2245.8	223.8 ± 24.7	2.7 ± 3.4	1.4 ± 0.1	4.2 ± 5.4	0.5 ± 0.1	0 ± 0	0 ± 0
	Day-21	5452.3 ± 4560.8	1360.3 ± 1848.3	79.6 ± 81.6	3.1 ± 0.1	13.2 ± 11	3.3 ± 4.5	0.2 ± 0.2	0 ± 0
	Day-28	8337.3 ± 5458.9	3237.3 ± 480.8	21.5 ± 0.6	4.9 ± 1.4	20.2 ± 13.2	7.8 ± 1.2	0.1 ± 0	0 ± 0
**Zn**	Day-7	7878.7 ± 3153.7	5143.7 ± 106.1	100.7 ± 7.1	330.5 ± 85.6	4.4 ± 1.8	2.9 ± 0.1	0.1 ± 0	0.2 ± 0
	Day-21	17158.7 ± 6576.1	6423.7 ± 4617.4	3903.7 ± 3712.3	575.5 ± 116.7	9.6 ± 3.7	3.6 ± 2.6	2.2 ± 2.1	0.3 ± 0.1
	Day-28	25808.7 ± 10323.8	13558.7 ± 1202.1	9653.7 ± 2057.7	568.5 ± 235.5	14.5 ± 5.8	7.6 ± 0.7	5.4 ± 1.2	0.3 ± 0.1
**Co**	Day-7	735 ± 0	571 ± 2.8	1.1 ± 0	41.3 ± 7.4	17.4 ± 0	13.5 ± 0.1	0 ± 0	1 ± 0.2
	Day-21	1022.5 ± 109.6	753.5 ± 70	823.5 ± 163.3	60.9 ± 7.6	24.1 ± 2.6	17.8 ± 1.7	19.4 ± 3.9	1.4 ± 0.2
	Day-28	1210 ± 169.7	952.5 ± 6.4	1025 ± 35.4	56 ± 20.2	28.6 ± 4	22.5 ± 0.2	24.2 ± 0.8	1.3 ± 0.5
**As**	Day-7	196 ± 45.3	274 ± 14.1	<10	5.5 ± 0	0.5 ± 0.1	0.7 ± 0	0 ± 0	0 ± 0
	Day-21	532 ± 67.9	383.5 ± 200.1	154 ± 11.3	21.6 ± 0.6	1.4 ± 0.2	1 ± 0.5	0.4 ± 0	0.1 ± 0
	Day-28	1640 ± 636.4	1673 ± 108.9	295 ± 66.5	34 ± 10.4	4.3 ± 1.7	4.4 ± 0.3	0.8 ± 0.2	0.1 ± 0
**Cd**	Day-7	41.2 ± 0.3	36.6 ± 0.5	<1	3.6 ± 0.6	9.2 ± 0.1	8.1 ± 0.1	0 ± 0	0.8 ± 0.1
	Day-21	65.3 ± 15.1	44.6 ± 5.1	47.2 ± 7.3	5.5 ± 1.1	14.5 ± 3.4	9.9 ± 1.1	10.5 ± 1.6	1.2 ± 0.2
	Day-28	87.2 ± 25.2	56.3 ± 4.3	61.1 ± 1.1	6.3 ± 2.4	19.4 ± 5.6	12.5 ± 1	13.6 ± 0.3	1.4 ± 0.5
**K**	Day-7	16500 ± 0	12500 ± 9899.5	62000 ± 16263.5	15250 ± 636.4	2.3 ± 0	1.7 ± 1.4	8.6 ± 2.2	2.1 ± 0.1
	Day-21	69500 ± 5656.9	58000 ± 2121.3	50500 ± 5656.9	17250 ± 1060.7	9.6 ± 0.8	8 ± 0.3	7 ± 0.8	2.4 ± 0.1
	Day-28	70500 ± 9899.5	59000 ± 2121.3	72500 ± 11313.7	18550 ± 3747.7	9.7 ± 1.4	8.2 ± 0.3	10 ± 1.6	2.6 ± 0.5
**Sb**	Day-7	26.9 ± 2.3	26.8 ± 0.4	<1	16 ± 1.1	1.1 ± 0.1	1.1 ± 0	0 ± 0	0.7 ± 0
	Day-21	46 ± 6.2	32.2 ± 0.4	30.4 ± 1.2	14.4 ± 2	1.9 ± 0.3	1.3 ± 0	1.3 ± 0.1	0.6 ± 0.1
	Day-28	61 ± 10.8	40.8 ± 9.4	37.1 ± 3.3	14.6 ± 2.1	2.6 ± 0.5	1.7 ± 0.4	1.6 ± 0.1	0.6 ± 0.1
**Ag**	Day-7	<5	<5	<5	<5	0 ± 0	0 ± 0	0 ± 0	0 ± 0
	Day-21	3.8 ± 0.7	4.1 ± 1.1	9.2 ± 3.7	<1	1.3 ± 0.2	1.4 ± 0.4	3.2 ± 1.3	0 ± 0
	Day-28	9.9 ± 4.6	13.3 ± 5.4	13.7 ± 6.4	<1	3.5 ± 1.6	4.7 ± 1.9	4.8 ± 2.2	0 ± 0
**Mn**	Day-7	3457.3 ± 56.6	2777.3 ± 56.6	4.5 ± 0.1	1670 ± 240.4	9.5 ± 0.2	7.7 ± 0.2	0 ± 0	4.6 ± 0.7
	Day-21	4462.3 ± 275.8	3452.3 ± 120.2	3842.3 ± 502	2285 ± 247.5	12.3 ± 0.8	9.5 ± 0.3	10.6 ± 1.4	6.3 ± 0.7
	Day-28	4747.3 ± 480.8	3922.3 ± 247.5	5102.3 ± 445.5	2400 ± 721.2	13.1 ± 1.3	10.8 ± 0.7	14.1 ± 1.2	6.6 ± 2

Table S9. Concentrations and percentages of metal(loid)s extracted from the Neves Corvo waste rock (NC_01) by the second experiment with *T. pacifica* via one-step bioleaching (1SB), two-step bioleaching (2SB), spent medium bioleaching (SMB), and Abiotic control (AC). Values are the average of duplicate flasks ± standard deviations.

		Concentration (μg/L)				% Recovery			
**Metal(loid)s**	**Days**	**1SB**	**2SB**	**SMB**	**AC**	**1SB**	**2SB**	**SMB**	**AC**
**Cu**	Day-7	1259.1 ± 56.6	1804.1 ± 106.1	1289.1 ± 169.7	7.2 ± 0.5	1.4 ± 0.1	2 ± 0.1	1.4 ± 0.2	0 ± 0
	Day-21	8534.1 ± 91.9	8154.1 ± 502	4159.1 ± 155.6	15.6 ± 4	9.3 ± 0.1	8.9 ± 0.5	4.5 ± 0.2	0 ± 0
	Day-28	12089.1 ± 424.3	9834.1 ± 360.6	7309.1 ± 905.1	16.8 ± 0.8	13.1 ± 0.5	10.7 ± 0.4	7.9 ± 1	0 ± 0
**Pb**	Day-7	19.2 ± 5.3	21.9 ± 1.1	61.2 ± 11.6	3.3 ± 1.5	0 ± 0	0.1 ± 0	0.1 ± 0	0 ± 0
	Day-21	5397.3 ± 84.9	4452.3 ± 247.5	4167.3 ± 410.1	<1	13.1 ± 0.2	10.8 ± 0.6	10.1 ± 1	0 ± 0
	Day-28	6912.3 ± 289.9	4032.3 ± 134.4	<1	4592.3 ± 657.6	16.7 ± 0.7	9.8 ± 0.3	11.1 ± 1.6	0 ± 0
**Zn**	Day-7	595.7 ± 159.8	677.7 ± 72.1	3013.7 ± 261.6	187.5 ± 91.2	0.3 ± 0.1	0.4 ± 0	1.7 ± 0.1	0.1 ± 0.1
	Day-21	26058.7 ± 212.1	27558.7 ± 1626.3	15908.7 ± 0	144.5 ± 3.5	14.6 ± 0.1	15.5 ± 0.9	8.9 ± 0	0.1 ± 0
	Day-28	26058.7 ± 212.1	27558.7 ± 1626.3	15908.7 ± 0	144.5 ± 3.5	17.5 ± 0.6	16.4 ± 0.6	12.2 ± 0.8	0.2 ± 0
**Co**	Day-7	470 ± 0	557 ± 31.1	824.5 ± 27.6	33.2 ± 10	11.1 ± 0	13.2 ± 0.7	19.5 ± 0.7	0.8 ± 0.2
	Day-21	1315 ± 21.2	1360 ± 84.9	1165 ± 21.2	22.5 ± 0.3	31.1 ± 0.5	32.1 ± 2	27.5 ± 0.5	0.5 ± 0
	Day-28	1415 ± 49.5	1355 ± 49.5	1355 ± 91.9	39.9 ± 0.2	33.4 ± 1.2	32 ± 1.2	32 ± 2.2	0.9 ± 0
**As**	Day-7	249 ± 9.9	341 ± 1.4	232.5 ± 19.1	<10	0.6 ± 0	0.9 ± 0	0.6 ± 0	0 ± 0
	Day-21	1480 ± 0	1550 ± 99	576 ± 148.5	<10	3.9 ± 0	4 ± 0.3	1.5 ± 0.4	0 ± 0
	Day-28	1325 ± 35.4	756 ± 21.2	1166 ± 712.8	<10	3.5 ± 0.1	2 ± 0.1	3 ± 1.9	0 ± 0
**Cd**	Day-7	28.4 ± 0.8	30.3 ± 0.7	43.8 ± 1.7	2.8 ± 0.9	6.3 ± 0.2	6.7 ± 0.2	9.7 ± 0.4	0.6 ± 0.2
	Day-21	92.6 ± 0.8	100.1 ± 5.6	63.3 ± 2.1	2.4 ± 0.1	20.6 ± 0.2	22.2 ± 1.2	14.1 ± 0.5	0.5 ± 0
	Day-28	105.5 ± 4.9	101.4 ± 5.1	80.9 ± 6.4	4.7 ± 0.2	23.4 ± 1.1	22.5 ± 1.1	18 ± 1.4	1.1 ± 0.1
**K**	Day-7	42500 ± 1414.2	33500 ± 14142.1	64500 ± 21213.2	15650 ± 353.6	5.9 ± 0.2	4.6 ± 2	8.9 ± 2.9	2.2 ± 0
	Day-21	108500 ± 5656.9	120500 ± 24041.6	141500 ± 29698.5	18850 ± 636.4	15 ± 0.8	16.7 ± 3.3	19.6 ± 4.1	2.6 ± 0.1
	Day-28	98500 ± 16970.6	70500 ± 14142.1	161500 ± 48083.3	17350 ± 353.6	13.6 ± 2.3	9.7 ± 2	22.3 ± 6.6	2.4 ± 0
**Sb**	Day-7	39.4 ± 1.6	48.5 ± 1.8	39.2 ± 0.9	13.1 ± 0.1	1.6 ± 0.1	2 ± 0.1	1.6 ± 0	0.5 ± 0
	Day-21	94.5 ± 1.1	105.5 ± 6.4	64.9 ± 12.4	13.2 ± 0	4 ± 0	4.4 ± 0.3	2.7 ± 0.5	0.6 ± 0
	Day-28	119 ± 5.7	119.5 ± 4.9	89.9 ± 22.8	12 ± 0.1	5 ± 0.2	5 ± 0.2	3.8 ± 1	0.5 ± 0
**Ag**	Day-7	<1	<1	4.5 ± 0.2	<1	0 ± 0	1.6 ± 0.1	0 ± 0	0 ± 0
	Day-21	28.9 ± 1	27.3 ± 1.7	21.4 ± 1.4	<1	10.1 ± 0.3	9.6 ± 0.6	7.5 ± 0.5	0 ± 0
	Day-28	24.8 ± 1.3	33.9 ± 1.6	36 ± 2.5	<1	8.7 ± 0.4	11.9 ± 0.6	12.6 ± 0.9	0 ± 0
**Mn**	Day-7	2807.3 ± 297	2257.3 ± 141.4	4282.3 ± 77.8	1595 ± 205.1	7.8 ± 0.8	6.2 ± 0.4	11.8 ± 0.2	4.4 ± 0.6
	Day-21	5282.3 ± 63.6	4867.3 ± 282.8	5052.3 ± 120.2	1315 ± 21.2	14.6 ± 0.2	13.4 ± 0.8	14 ± 0.3	3.6 ± 0.1
	Day-28	4862.3 ± 176.8	4347.3 ± 155.6	5727.3 ± 0	1880 ± 14.1	13.4 ± 0.5	12 ± 0.4	15.8 ± 0	5.2 ± 0
